# Springtime soil and tree stem greenhouse gas fluxes and the related soil microbiome pattern in a drained peatland forest

**DOI:** 10.1007/s10533-025-01238-3

**Published:** 2025-05-07

**Authors:** Reti Ranniku, Fahad Ali Kazmi, Mikk Espenberg, Joosep Truupõld, Jordi Escuer-Gatius, Ülo Mander, Kaido Soosaar

**Affiliations:** 1 Department of Geography, Institute of Ecology & Earth Sciences, University of Tartu, 46 Vanemuise, EST-51014 Tartu, Estonia; 2 Institute of Agricultural and Environmental Sciences, Estonian University of Life Sciences, 5 Fr.R. Kreutzwaldi, EST-51006 Tartu, Estonia

**Keywords:** Carbon dioxide, Denitrification, Functional genes, Methane, Nitrous oxide, Stem fluxes

## Abstract

Spring can be a critical time of year for stem and soil methane (CH_4_), nitrous oxide (N_2_O) and carbon dioxide (CO_2_) emissions as soil freeze–thaw events can be hot moments of gas release. Greenhouse gas fluxes from soil, Downy birch (*Betula pubescens*) and Norway spruce (*Picea abies*) stems were quantified using chamber systems and gas analysers in spring 2023 in a northern drained peatland forest. Dissolved gas concentrations in birch sap and soil water, environmental parameters, soil chemistry, and functional gene abundances in the soil were determined. During spring, initially low soil and stem CH_4_, N_2_O, and CO_2_ emissions increased towards late April. Temperature emerged as the primary driver of soil and stem fluxes, alongside photosynthetically active radiation influencing stem fluxes. Soil hydrologic conditions had minimal short-term impact. No clear evidence linked stem CH_4_ emissions to birch sap gas concentrations, while relationships existed for CO_2_. Functional gene abundances of the N and CH_4_-cycles changed between measurement days. Potential for methanogenesis and complete denitrification was higher under elevated soil water content, shifting to methanotrophy and incomplete denitrification as the study progressed. However, our results highlight the need for further analysis of relationships between microbial cycles and GHG fluxes under different environmental conditions, including identifying soil microbial processes in soil layers where tree roots absorb water.

**Supplementary Information:**

The online version contains supplementary material available at 10.1007/s10533-025-01238-3.

## Introduction

Peatlands are a key ecosystem regulating global climate due to the crucial role of peatland soils in storing carbon (C) and nitrogen (N). Peatland drainage for agriculture or forestry can disrupt the natural hydrological balance of peatlands, lowering the groundwater table, switching the soil conditions from anaerobic to aerobic. Consequentially, peatland soils can shift from a carbon dioxide (CO_2_) sink to a source, while methane (CH_4_) emissions are supressed and nitrous oxide (N_2_O) release is increased (Korkiakoski et al. 2019; Pihlatie et al. 2010). Continuous peatland drying is expected to have a net global warming effect due to increased CO_2_ emissions surpassing reduced CH_4_ emissions (Huang et al. 2021). Tree stems play a vital role in regulating greenhouse gas (GHG) balances in forested peatlands. Stems of various tree species have been shown to exchange GHGs with the atmosphere. Despite the established importance of stem fluxes, their incorporation into GHG models and assessments is frequently overlooked, primarily due to the challenges associated with spatiotemporal variability and uncertainty in flux dynamics, and the complexities in upscaling fluxes to the entire ecosystem (Barba et al. 2024; Jeffrey et al. 2023b; Machacova et al. 2019).

Soil hydrology is the key factor driving peatland soil GHG dynamics. Thus, disturbances in hydrological regimes, for example due to climate warming, can significantly impact peatland soil C and N retention (Hugelius et al. 2020). Increased and higher intensity precipitation can lead to a greater frequency of flood events, which can elevate stem CH_4_ and N_2_O release in riparian forests (Schindler et al. 2020). Wet period stem CH_4_ emissions contribute substantially more to ecosystem fluxes compared to drier periods (Mander et al. 2022). Furthermore, spring freeze–thaw cycles have been shown to be crucial in regulating GHG dynamics in forested peatlands. Fluctuations in the water table associated with freeze–thaw events can induce hot moments of N_2_O emissions from both soil (Mander et al. 2021) and tree stems (Ranniku et al. 2023). Climate change may increase the frequency of freeze–thaw events in northern latitudes (Henry 2008), emphasising the importance of studying springtime soil and stem GHG dynamics to better understand the underlying processes during this critical period.

Various biophysical processes regulate soil and tree stem GHG fluxes in drained peatlands. Production and consumption of CH_4_ co-occur in the soil, determining the net flux at the soil surface (Ni & Groffman 2018). CH_4_ production in soil arises from anaerobic microbial methanogenesis, a process carried out by archaea possessing the *mcrA* gene which encodes the methyl-coenzyme M reductase (MCR) enzyme complex (Borrel et al. 2019). CH_4_ is consumed under aerobic conditions by methanotrophs harbouring the *pmoA* gene, encoding the particulate CH_4_ monooxygenase enzyme (pMMO) essential for CH_4_ oxidation (Hanson & Hanson 1996; Veldkamp et al. 2013), and through nitrate-dependent anaerobic methane oxidation (n-damo) (Ettwig et al. 2010; Haroon et al. 2013; Hu et al. 2014). N_2_O is produced in the soil through microbial nitrification and denitrification (Butterbach-Bahl et al. 2013). Nitrification occurs aerobically, where nitrifier microbes carrying the *amoA* gene oxidise ammonia to nitrite and nitrate. In contrast, denitrification occurs anaerobically, driven by microbes possessing the *nirS* and *nirK* genes, which reduce nitrates to N gases, including N_2_O. The denitrification process is completed by denitrifiers carrying the Clade I or II *nosZ* genes, converting N_2_O to N_2_ gas (Kuypers et al. 2018). Hence, if this consumption of N_2_O by denitrifiers is slower than its production, N_2_O can accumulate, contributing to N_2_O emissions from soil (Braker & Conrad 2011). CO_2_ is released from the soil through autotrophic respiration by plant roots, divided into maintenance, growth and ion uptake respiration (Hirano et al. 2023), as well as through heterotrophic respiration resulting from the decomposition of soil organic matter and fauna by microorganisms (Schindlbacher et al. 2009).

Tree stem fluxes can be produced both in the soil and within the stem itself. However, a comprehensive understanding of the factors that determine the net flux remains lacking. Soil-produced CH_4_, N_2_O and CO_2_ are dissolved in soil water and taken up by plant roots. Gases move up the xylem due to a negative pressure gradient caused by leaf transpiration (Venturas et al. 2017). Eventually, these gases diffuse into the atmosphere due to a concentration gradient between the stem and the surrounding air or transpire to the atmosphere through the leaves. Moreover, previous studies have identified microbial CH_4_ production (Barba et al. 2019a; Pitz & Megonigal 2017; Putkinen et al. 2021) and consumption (Jeffrey et al. 2021; Putkinen et al. 2021) within tree stems, as well as N_2_O production (Lenhart et al. 2015) and consumption (Machacova et al. 2017) from cryptogamic covers on stem bark. Additionally, stem CO_2_ efflux can be influenced by photosynthesis and stem respiration (Gansert & Burgdorf 2005; Salomón et al. 2021). Partitioning of the precise origin of stem-emitted GHGs continues to be a key area of research.

Interactions between various environmental factors and processes govern the exchange of GHGs between the soil, stems, and the atmosphere. Gas production and their solubility in the soil are primarily controlled by groundwater table depth (WTD), soil water content (SWC), soil temperature (T_soil_) and nutrient availability (Barba et al. 2019a; Pitz & Megonigal 2017; Teskey et al. 2008). The density of the root system determines the extent to which dissolved gases are absorbed by plant roots (Bachofen et al. 2024; Puhe 2003), and the xylem sap flow rate dictates gas transport within tree stems (Gansert & Burgdorf 2005). Additionally, stem morphology and tree physiological traits, such as wood density and lenticel abundance, play a critical role in controlling the eventual gas diffusion into the atmosphere (Pangala et al. 2013; Pitz et al. 2018; Teskey et al. 2008).

The xylem sap within birch trees, such as downy birch (*Betula pubescens* Ehrh.), experiences positive pressures during springtime in northern regions, leading to sap exudation from cuts or holes in the tree stem (Hölttä et al. 2018). This phenomenon generally occurs after soil thaw, lasting until budburst, when pressures in the xylem turn negative again due to transpiration from the leaves. Coupled with the previously observed increased birch stem emissions during the freeze–thaw periods (Ranniku et al. 2023), spring in these regions provides an opportunity to study the processes related to birch stem flux dynamics during a period of xylem sap pressure changes (Hölttä et al. 2018).

Therefore, this study aimed to quantify CH_4_, N_2_O and CO_2_ fluxes from soil and tree stems, and to assess whether changes in soil chemical and microbiological conditions can influence fluxes during springtime under conditions following the freeze–thaw period, in a drained peatland forest. Furthermore, the relationships between CH_4_ and CO_2_ fluxes from Downy birch stems and the concentrations of dissolved CH_4_ and CO_2_ within the birch sap were explored. Thus, it was hypothesised that (I) soil and birch stem CH_4_ and N_2_O fluxes are higher under elevated SWC, with soil water parameters driving the fluxes, while spruce stem fluxes remain low throughout the study period; (II) higher dissolved gas concentrations in birch sap correspond to higher birch stem GHG fluxes; (III) soil microbial N and CH_4_-cycling community composition changes together with changes in SWC, with elevated abundances of denitrifying and methanogenic microbes under higher SWC conditions.

## Methods

### Site description and study design

The experiment was carried out in a drained peatland forest site (58°17′N, 27°17′E; 38 m.a.s.l.; 1.72 ha; Supplementary Fig. S1) in eastern Estonia, belonging to the warm summer humid continental climate zone (Köppen 1936), and the hemiboreal vegetation zone (Ahti et al. 1968). The region receives an average annual precipitation of 650 mm, with mean temperatures of 17 °C in July and − 6.7 °C in January, and a growing season that lasts 175–180 days (Kupper et al. 2011). The site was drained roughly 50–55 years prior to the study using an open-ditch drainage system. Drainage ditches (~ 2 m width) surround the study site on all sides, with the western ditch being a small river (Supplementary Fig. S1). The primary tree species in the forest are downy birch (*Betula pubescens* Ehrh.) and Norway spruce (*Picea abies* (L.) H. Karst.). Tree stand characteristics have been brought out in Supplementary Table S2. The soil is classified as Drainic Eutric Histosol (IUSS Working Group WRB, 2015), featuring a 100 cm peat layer depth, low dry bulk density, and high N and organic C contents (Uri et al. 2017). Soil gas concentration measurements and soil sampling were performed from 12 monitoring points within a 50 × 70 m study plot, located within the larger study area. The study plot was located roughly 100 m from the southern drainage ditch, 90 m from the western ditch, 85 m from the eastern ditch and 495 m from the northern ditch. Stem gas concentration measurements were performed from six downy birch trees, as well as five Norway spruce trees for comparison, located adjacent to the soil chambers (Supplementary Fig. S1).

### GHG flux measurements

The study period (06/04/2023–12/05/2023) ran from thawing of the frozen soil until budburst, together with the first weeks of the vegetation period. A total of 11 sampling campaigns were conducted for tree stem gas sampling, with measurements twice per week. Sampling was performed from stem chamber systems, with two chambers per tree height, distributed across 180°, covering 0.0108 m^2^ of the stem surface and 0.00119 m^3^ in volume. Stem chambers were constructed from transparent rectangular plastic containers (Lock & Lock, Seoul, South Korea) with their bottoms removed. Chambers were affixed to the stem surface at approximately 10 cm, 80 cm, and 170 cm, depending on the physical characteristics of each tree and the feasibility of installation, to capture the vertical profile of stem flux. During the 5-min measurement time, the stem chambers were closed airtightly with lids. The gas concentrations in the chamber systems were monitored using trace gas analysers (LI-7810 for CH_4_ and CO_2_ and LI-7820 for N_2_O; Li-Cor Biosciences, Lincoln, NE, USA), which were connected to the lids with nylon tubing, circulating air in a closed loop between the chamber and the analyser (Supplementary Fig. S2).

Soil GHG flux measurements were performed continuously during the study period (06/04/2023—12/05/2023) using automated dynamic chambers at each monitoring point, each covering an area of 0.16 m^2^ soil surface and encompassing 0.032 m^3^ in volume. A multiplexer facilitated automated continuous measurements, whereby chambers closed for nine minutes each, followed by flushing with ambient air. During closure, chamber air was analysed using a gas analyser (G2508, Picarro Inc., Santa Clara, California, United States), employing cavity ring-down spectroscopy to quantify CO_2_, CH_4_ and N_2_O concentrations. The 12 soil chambers completed one full measurement cycle every 2 h, yielding 12 flux measurements per chamber per day.

Soil and stem CO_2_, CH_4_ and N_2_O fluxes were quantified using the ideal gas formula, based on the linear regression of changes in chamber gas concentrations over time. Detailed equations used for the calculation can be found in Ranniku et al. (2024). The last 180 s of the total five-minute stem flux measurement time, and the middle 300 s of the nine-minute soil flux measurement time were used for calculations to eliminate the initial stabilisation period.

The quality of chamber closure was verified by the adjusted R^2^ value from the linear regression of CO_2_ measurements. Fluxes with an R^2^ value above 0.9 were accepted, and the flagged measurements were further visually inspected to determine potential biases of removing low fluxes. Low-flux measurements showing acceptable concentration change patterns during visual inspection were retained in the analysis to avoid systematic exclusion of valid low-flux data. This filtering led to removal of 24.1% of soil flux, 0.51% of birch stem flux, and 3.13% of spruce flux measurement sessions. Visual inspection of the filtered data revealed that the high percentage of soil flux removal was primarily due to chamber closure issues, particularly occasional snow obstruction that hindered proper chamber sealing. However, the discarded data were distributed relatively uniformly across the study period, with no systematic biases observed.

### Dissolved gas concentrations in birch sap and soil water

The dissolved CH_4_ and CO_2_ concentrations in birch sap (dCH_4sap_ and dCO_2sap_) and soil water (dCH_4soil_ and dCO_2soil_) were determined. The N_2_O measurements could not be obtained due to a technical malfunction. Birch sap was collected in syringes connected to holes tapped into the stems of six birch trees at 10 cm and 170 cm heights. Birch sap was collected between 17/04/2023–04/05/2023, but the exudation period varied between individual trees. For the water-atmosphere equilibration, 30 ml of birch sap was collected in the syringe and 30 ml of ambient air was added to the headspace. To determine dCH_4soil_ and dCO_2soil_, water samples were collected into syringes from the water table surface in groundwater wells at each monitoring point. The syringe was then shaken for one minute (Magen et al. 2014; Sapper et al. 2023), and the mixed headspace air was pushed to pre-evacuated gas-tight vials, taken to the laboratory to be analysed with gas chromatography (GC-2014, Shimadzu, Kyoto, Japan).

The dissolved CH_4_ and CO_2_ (µmol L^−1^) concentrations in birch sap and soil water were calculated based on Magen et al. (2014), as the sum of dissolved gas in the syringe headspace and dissolved gas remaining in the water after shaking. The headspace CH_4_ and CO_2_ concentrations in ppm were converted to gas amount using the ideal gas law at the temperature of sample extraction (10 °C).

1 $${D}_{gas}= \frac{HS\times {V}_{hs}\times P}{R\times {T\times V}_{water}}\times \left(1+\upbeta \frac{{V}_{water}}{{V}_{hs}}\right) \left[\mu mol {L}^{-1}\right],$$

where D_gas_ is the dissolved CH_4_ or CO_2_ concentrations in birch sap or soil water in µmol L^−1^, HS is the mole fraction of headspace CH_4_ or CO_2_ in ppm, V_hs_ is the volume of the syringe headspace (0.03 L), P is the atmospheric pressure (1 atm), R is the ideal gas constant (0.08206 atm L K^−1^ mol^−1^), T is the air temperature at sample extraction (283.15 °K), V_water_ is the volume of water in the syringe (0.03 L) and β is the Bunsen coefficient for 10 °C at 0 salinity (β_CH4_ = 0.04417 according to Yamamoto et al. (1976), β_CO2_ = 0.05366 according to Weiss (1974)).

### Meteorological and soil physical parameters

Air (T_air_; Rotronic HC2A-S3; Rotronic AG, Bassersdorf, Switzerland) and soil temperature (T_soil_; 107, CAMPBELL SCIENTIFIC. INC, Logan, Utah, USA), photosynthetically active radiation (PAR; LI-190SL; LI-COR Biosciences, Lincoln, NE, USA) and SWC (ML3 ThetaProbe, Delta-T Devices, Cambridge, United Kingdom) were continuously measured during the whole study period. T_soil_ and SWC sensors were placed at 10 cm soil depth next to the soil chambers. T_air_ and PAR were measured in a micrometeorological tower on site, at 5 m for T_air_ and 25 m for PAR (above the forest canopy). WTD was manually measured during each sampling day in groundwater wells adjacent to the soil chambers.

### Soil sampling and chemical analysis

Soil sample collection was performed weekly adjacent to each soil chamber from 0–10 cm belowground with a soil corer (n = 48). Three soil cores were collected and pooled from each sampling point to form a composite sample. Soil pH was analysed from a 1 M KCl solution. Ammonium (NH_4_^+^ − N) and nitrate (NO_3_^−^ − N) contents were measured from a 2 M KCl extract (1:10 ratio) of each soil sample by flow injection analysis (APHA-AWWA-WEF 2005). Total N and C contents in air-dried samples were quantified using the high-temperature combustion method on a Skalar Primacs SNC-100 elemental analyser (Skalar Analytical B.V., Breda, The Netherlands). All the soil chemical analyses were conducted in the Estonian University of Life Sciences soil laboratory.

### Soil DNA extraction and quantitative polymerase chain reaction

The soil microbial community composition was determined from soil samples collected on three dates (17/04/2023, 01/05/2023, 08/05/2023). DNA extraction was performed from 0.25 g soil sample with the DNeasy PowerSoil Pro kit (Qiagen, Hilden, Germany), following the manufacturer's protocol. Soil samples were homogenised using Precellys 24 Homogeniser (Berlin Technologies, Montigny-le-Bretonneux, France) at 5000 rpm for 20 s. The extracted DNA’s concentration and quality were determined with an Infinite M200 spectrophotometer (Tecan AG, Grodig, Austria). The DNA was kept at – 20 °C until further analyses.

The quantitative polymerase chain reaction (qPCR) assays were performed with RotorGene® Q equipment (Qiagen, Valencia, CA, USA). The abundance of bacterial and archaeal communities was assessed using bacterial and archaeal 16S rRNA genes. The functional genes for nitrification were determined through bacterial, archaeal, and COMAMMOX (complete ammonia oxidation) *amoA* genes, and for denitrification through *nirK* and *nirS* (nitrite reductase genes) and Clade I (*nosZ* I) and II (*nosZ* II) N_2_O reductase genes. The methanogenic *mcrA* gene and methanotrophic *pmoA* and *n-damo*-specific 16S rRNA genes were also quantified. Details of the gene-specific primer sets, optimised primer concentrations, and thermal cycling conditions for each target gene are shown in Espenberg et al. (2024). All qPCR measurements were conducted in replicates, with negative controls implemented to ensure no contamination. Gene copy numbers were calculated using standard curves, which were derived from serial dilutions of stock solutions containing target sequences (Eurofins MWG Operon, Germany). The qPCR data were processed in RotorGene Series Software (version 2.0.2, Qiagen, Hilden, Germany) and LinRegPCR program v. 2020.0. Gene abundances were calculated as the mean fold differences between samples and their respective tenfold standard dilution, as described in detail by Espenberg et al. (2018). Results were expressed as gene copies per gram of dry weight (gene copies g^−1^ dw). The DNA extraction and qPCR were conducted in the University of Tartu’s Department of Geography microbiology laboratory.

### Statistical analysis

Statistical analyses were conducted with R version 4.0.3 (R core team 2020). Data normality was evaluated with the Kolmogorov–Smirnov test. Since the flux data did not follow a normal distribution, non-parametric methods were applied. Temporal variability in gas fluxes and functional gene abundances, as well as differences in tree stem fluxes between different heights (10 cm, 80 cm, and 170 cm) were analysed using the Kruskal–Wallis one-way analysis of variance, followed by Dunn’s multiple comparison test with Bonferroni adjustment for post-hoc analysis. For birch stem CH_4_ and spruce stem CO_2_ fluxes, which showed a significant vertical gradient, linear interpolation was applied to estimate mean fluxes across all sampled heights to represent the overall stem flux. For other fluxes, where no significant vertical gradient was observed, the mean of the three height measurements was used.

Spearman's rank correlation was employed to examine correlations, and the linear regression model was applied to determine relationships between fluxes and control parameters. In addition, to further assess the effects of the main environmental parameters (T_soil_, SWC, WTD) on gas fluxes, a mixed-effects linear model was employed, accounting for the grouped structure of the data, including repeated measurements taken from subplots as random effects. Principal Component Analysis (PCA) was performed to visualise relationships between variables. Statistical significance was set at *p* < 0.05.

## Results

**Fig. 1:**
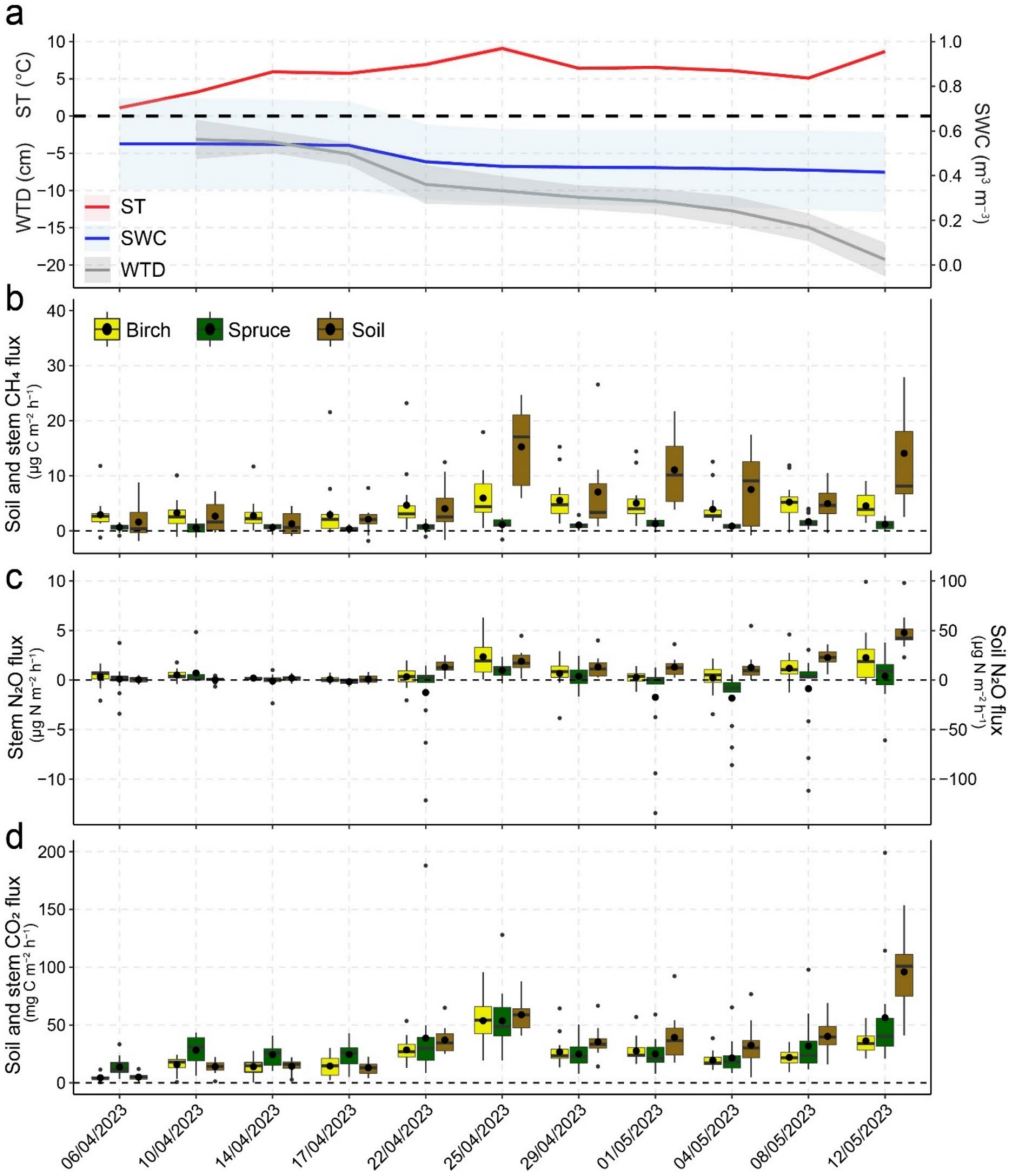
Temporal dynamics of environmental parameters, and soil and tree stem CH_4_, N_2_O and CO_2_ fluxes during the study period (06/04/2023–12/05/2023). **(a)** Daily mean soil temperature (T_soil_, ◦C), water table depth (WTD, cm), and soil water content (SWC, m^3^ m^–3^) with 95% confidence intervals as the shaded area; boxplots of soil and tree stem **(b)** CH_4_ (μg C m^–2^ h^–1^), **(c)** N_2_O (μg N m^–2^ h^–1^) and **(d)** CO_2_ (mg C m^–2^ h^–1^) fluxes. Median values are marked with solid lines within boxes, mean values with circles, 25th and 75th percentiles with box boundaries, maximum and minimum values with whiskers, outliers with dots (n = 12 for soil, n = 6 for birch, n = 5 for spruce on each day). Note the secondary axis for **(a)** SWC and **(c)** Soil N_2_O flux

The average (mean ± SE) environmental parameters during the measurement days on the study site were: T_air_ 10.3 ± 0.3 °C, T_soil_ 5.82 ± 0.10 °C, SWC 0.50 ± 0.002 m^3^ m^–3^, WTD –10.3 ± 0.5 cm, PAR 324.6 ± 30.4 µmol m^–2^ s^–1^. T_air_ and T_soil_ increased in the springtime, peaking on 25/04/2023, followed by a decline and rising again towards mid-May. Meanwhile, SWC remained relatively stable throughout the study period and WTD continuously declined (Fig. 1a).

### Temporal dynamics and drivers of stem and soil CH_4_, N_2_O and CO_2_ fluxes

**Fig. 2:**
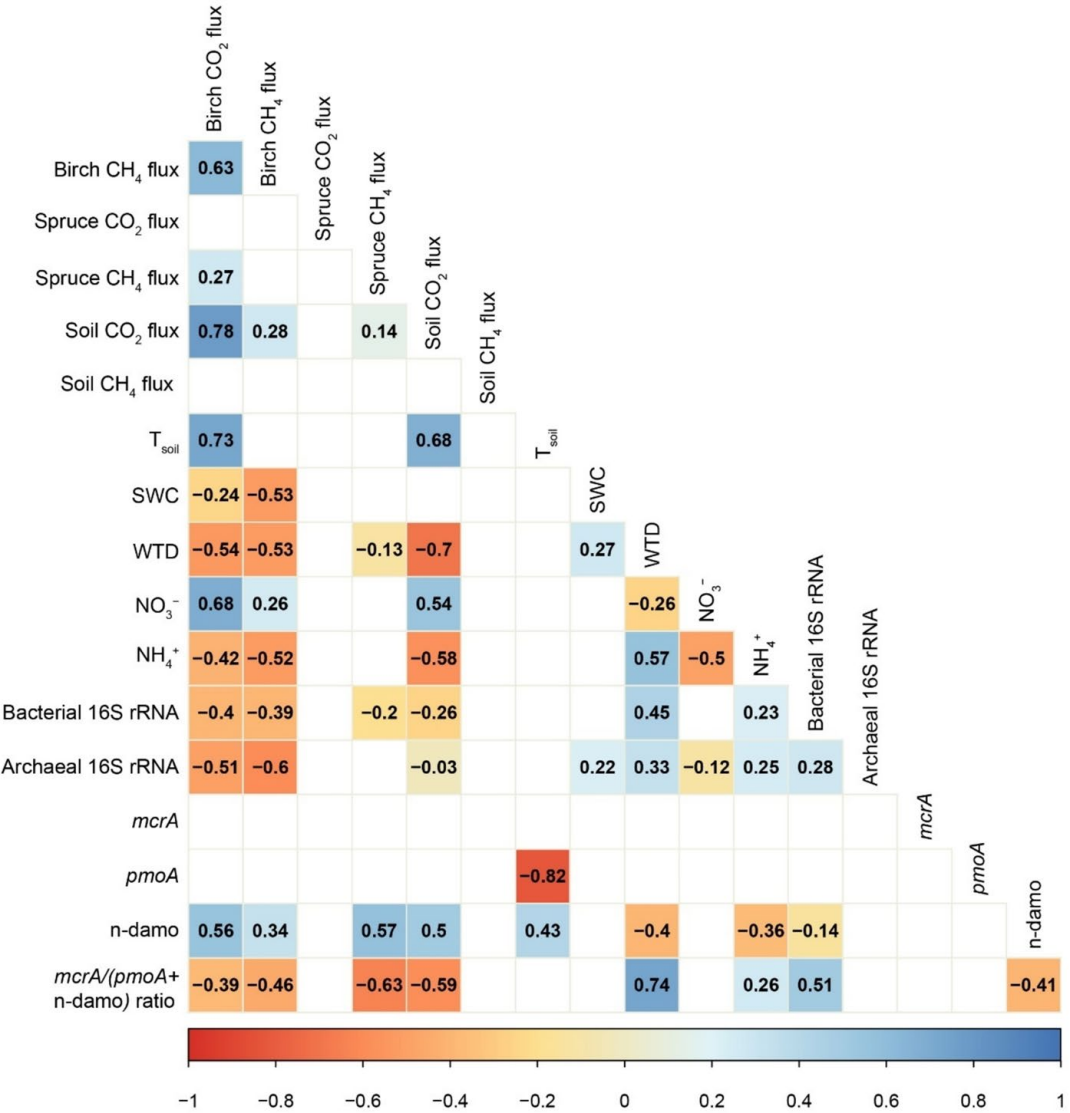
Correlation matrix for the CH_4_ cycle with Spearman’s rank correlation coefficients (ρ) between soil and stem CH_4_ and CO_2_ fluxes, CH_4_ cycling gene abundances, environmental parameters, and soil NO_3_ and NH_4_ contents. Insignificant correlations have been removed

Study period daily average birch, spruce and soil fluxes of all gases followed similar temporal trends. Fluxes remained low during the onset of spring, increasing at the end of April and again in May (Fig. 1b; 1c; 1d). Supplementary Fig. S3 also shows the continuous time series of soil fluxes, including days during the study period where stem fluxes were not measured. Daily average CH_4_ fluxes were 4.18 ± 0.33 µg CH_4_-C m^−‍2^ h^−‍1^ from birch stems, 0.918 ± 0.104 µg CH_4_-C m^−‍2^ h^−‍1^ from spruce stems, and 5.08 ± 1.38 µg CH_4_-C m^−‍2^ h^−‍1^ from soil. Fluxes had significant variation between tree species. Birch stem CH_4_ fluxes showed a statistically significant decline between 10 and 170 cm stem heights, as well as 80 cm and 170 cm (Supplementary Fig. S4). Spearman’s correlation and linear regression analysis showed that birch stem CH_4_ fluxes were significantly negatively correlated with SWC and WTD (Fig. 2), while being positively correlated to PAR (Supplementary Fig. S5). Spruce stem CH_4_ fluxes positively correlated with PAR and negatively to WTD. Soil CH_4_ fluxes had positive correlations with T_air_ (Supplementary Fig. S5). The mixed-effects linear model confirmed the negative relationship between birch and spruce CH_4_ fluxes and WTD and indicated a positive relationship between soil CH_4_ flux and T_soil_ (Supplementary Table S4).

**Fig. 3:**
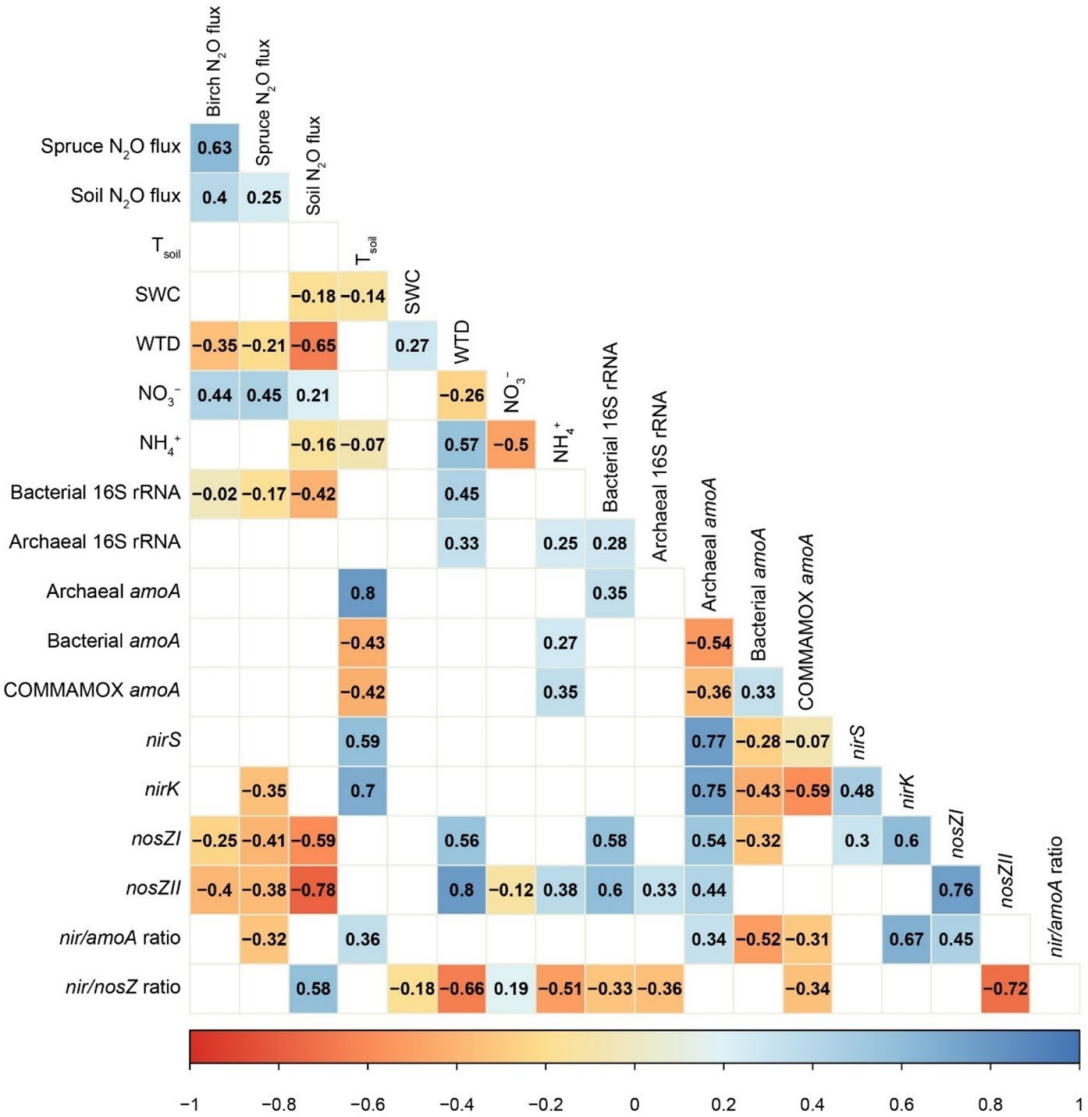
Correlation matrix for the N cycle with Spearman’s rank correlation coefficients between soil and stem N_2_O fluxes, N cycling gene abundances, environmental parameters, and soil NO_3_ and NH_4_ contents. Insignificant correlations have been removed

Stem N_2_O fluxes averaged at 0.778 ± 0.148 µg N_2_O-N m^−‍2^ h^−‍1^ from birch, –0.371 ± 0.329 µg N_2_O-N m^−‍2^ h^−‍1^ from spruce and 13.2 ± 1.4 µg N_2_O-N m^−‍2^ h^−‍1^ from soil. Fluxes had significant variation between tree species. Birch and soil N_2_O fluxes had positive correlations with T_air_ and PAR (Supplementary Fig. S5) and negative with WTD (Fig. 3). In addition, negative correlations occurred between spruce N_2_O fluxes and WTD, and soil N_2_O fluxes and SWC (Fig. 3). The mixed-effects model further indicated a significant positive influence of T_soil_ on birch and soil N_2_O fluxes, and negative influence of WTD on soil N_2_O fluxes (Supplementary Table S4).

Study-period average stem CO_2_ fluxes were 23.7 ± 1.8 mg CO_2_-C m^−‍2^ h^−‍1^ from birch, 33.1 ± 3.2 mg CO_2_-C m^−‍2^ h^−‍1^ from spruce, and 34.6 ± 2.5 mg CO_2_-C m^−‍2^ h^−‍1^ from soil. Fluxes had significant variation between tree species. Spruce CO_2_ fluxes displayed significant differences between fluxes at 10 cm and 170 cm stem heights (Supplementary Fig. S4). Spearman’s correlations and linear regression revealed that birch and soil CO_2_ fluxes correlated positively with T_soil_, T_air_ and PAR, and negatively with WTD (Fig. 2; Supplementary Fig. S5). Birch CO_2_ fluxes further demonstrated a negative correlation with SWC (Fig. 2). The mixed-effects model further showed that T_soil_ was a positive predictor of birch, spruce and soil CO_2_ fluxes, while WTD negatively influenced soil CO_2_ fluxes (Supplementary Table S4).

### Dissolved gas concentrations in birch sap and in soil water

**Fig. 4:**
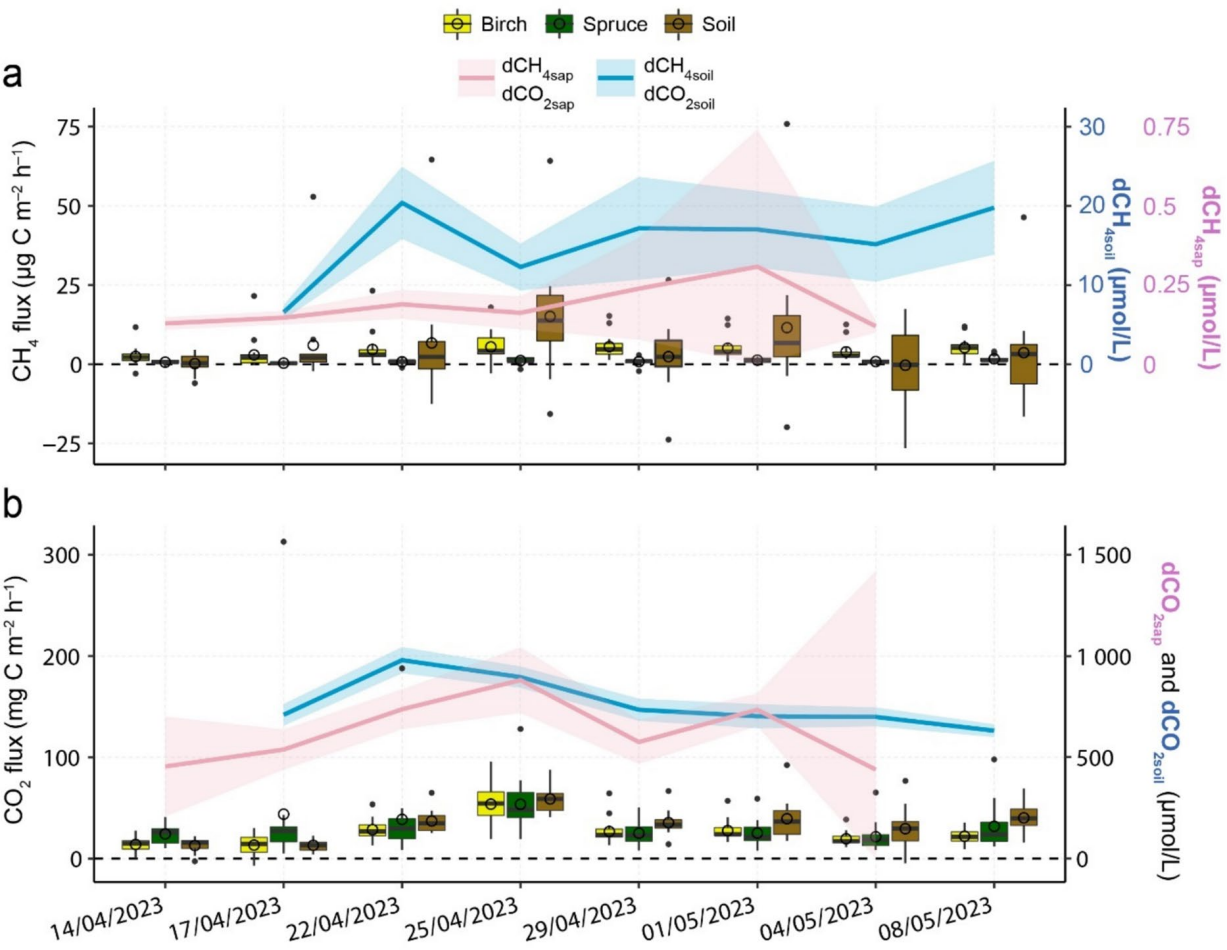
Temporal dynamics of soil and stem **(a)** CH_4_ fluxes (μg C m^–2^ h^–1^) and dissolved CH_4_ concentrations in birch sap (dCH_4sap_; µmol L^−1^) and in soil water (dCH_4soil_; µmol L^−1^), and **(b)** CO_2_ fluxes (mg C m^–2^ h^–1^) and dissolved CO_2_ concentrations in birch sap (dCO_2sap_; µmol L^−1^) and in soil water (dCO_2soil_; µmol L^−1^) during the birch sap sample collection dates (14/04/2023–04/05/2023) and soil water sample collection dates (17/04/2023–08/05/2023). Boxplots represent CH_4_ and CO_2_ fluxes, with median values across the measurement points marked by solid lines, mean values by circles, 25th and 75th percentiles by box boundaries and outliers by dots within each box (n = 12 for soil, n = 6 for birch, n = 5 for spruce on each day). Lines indicate daily mean values of the dissolved gas concentrations with 95% confidence intervals as the shaded area

While the emission of CH_4_ and CO_2_ from the stems of birch peaked on 25/04/2023, dCH_4sap_ was the highest on 01/05/2023 and dCO_2sap_ had higher concentrations on 25/04/2023 and 01/05/2023 (Fig. 4). Birch CH_4_ emissions were not significantly related to dCH_4sap_, while CO_2_ fluxes had significant correlations with dCO_2sap_, with the relationship being stronger on the lower part of the stem (Supplementary Fig. S6). There was no statistically significant difference between dCH_4sap_ and dCO_2sap_ at the measured stem heights of 10 and 170 cm.

Temporal trends of dCH_4soil_ and dCO_2soil_ showed that increased gas concentrations in soil water precede a peak in stem and soil fluxes (Fig. 4). However, only soil CH_4_ fluxes had significant correlations with dCH_4soil_ and spruce CO_2_ fluxes with dCO_2soil_. Spruce CH_4_ correlated negatively with dCH_4soil_ but spruce fluxes were negligible (Supplementary Fig. S6).

### Soil chemistry

Soil NH_4_ and NO_3_ contents exhibited opposing temporal trends (Supplementary Fig. S7). While the NO_3_ content showed similar temporal trends to GHG flux dynamics with a peak on 25/04/2023, NH_4_ content was elevated at the beginning of the measurement period, declining in the middle and slightly rising again at the end (Supplementary Fig. S7). Detailed soil chemical properties can be found in Supplementary Table S1.

Birch stem CH_4_ and CO_2_ fluxes, as well as soil CO_2_ and N_2_O, were negatively related to changes in the NH_4_ content in the soil, which was higher in the beginning of the study period and had itself a negative correlation with T_air_ and PAR, and positive with WTD (Supplementary Fig. S5). Conversely, soil NO_3_ content was positively correlated with fluxes, demonstrating significant relationships with birch fluxes of all gases, spruce N_2_O, and soil CO_2_ fluxes (Fig. 2; Fig. 3).

### Soil microbial composition

**Fig. 5:**
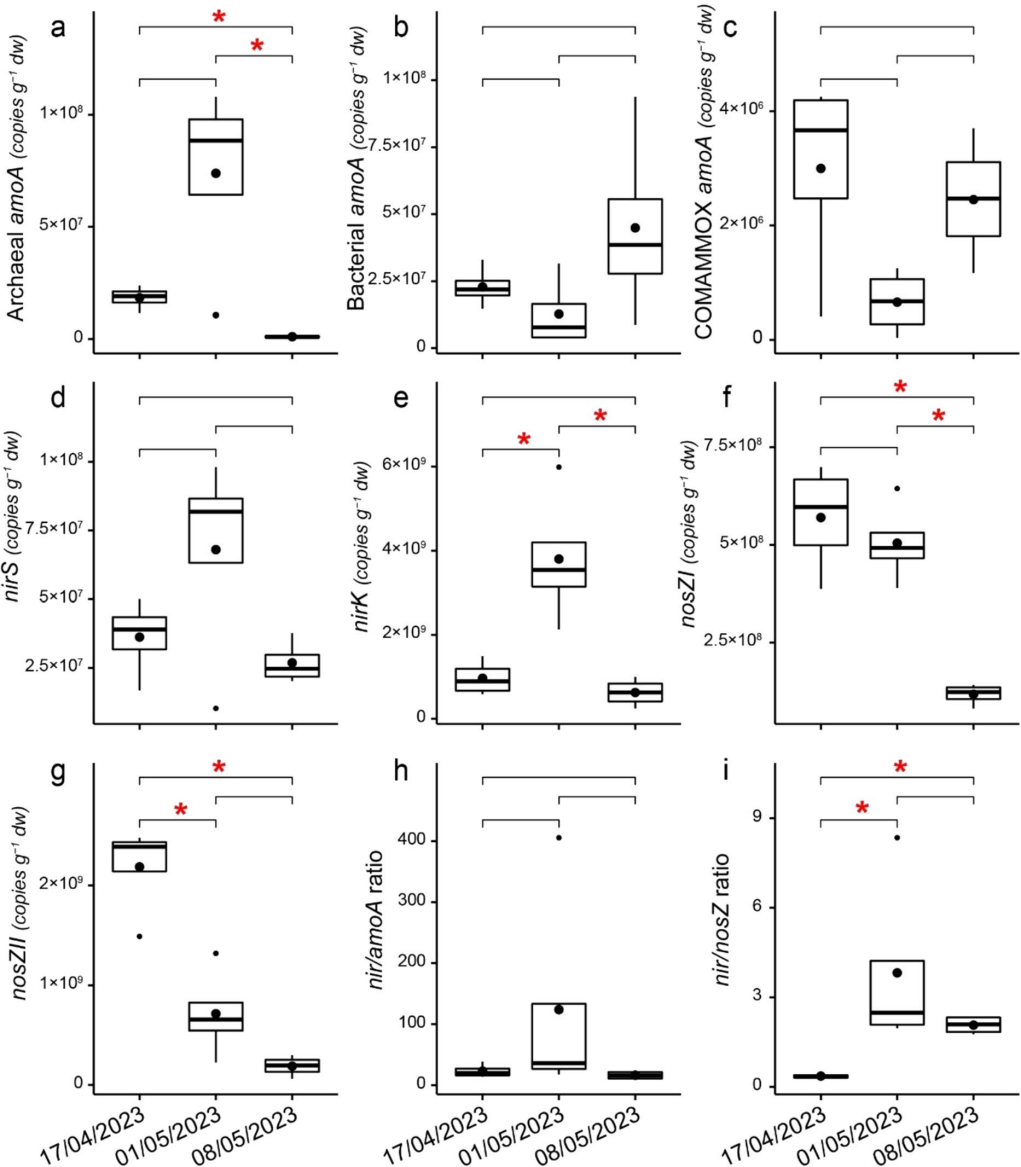
Boxplots of the N cycle functional gene abundances (gene copies per gram of dry soil) of **(a)** archaeal *amoA*, **(b)** bacterial *amoA*, **(c)** COMAMMOX *amoA*, **(d)** *nirS*, **(e)** *nirK*, **(f)** *nosZ* I, **(g)** *nosZ* II, and the **(h)** *nir*/*amoA* and **(i)** *nir*/*nosZ* gene ratios on soil sampling dates (17/04/2023, 01/05/2023, 08/05/2023). Median values are marked with solid lines, mean values with circles, 25th and 75th percentiles with box boundaries and outliers with dots. Asterisks above bars show statistically significant differences between gene abundances on different days

**Fig. 6:**
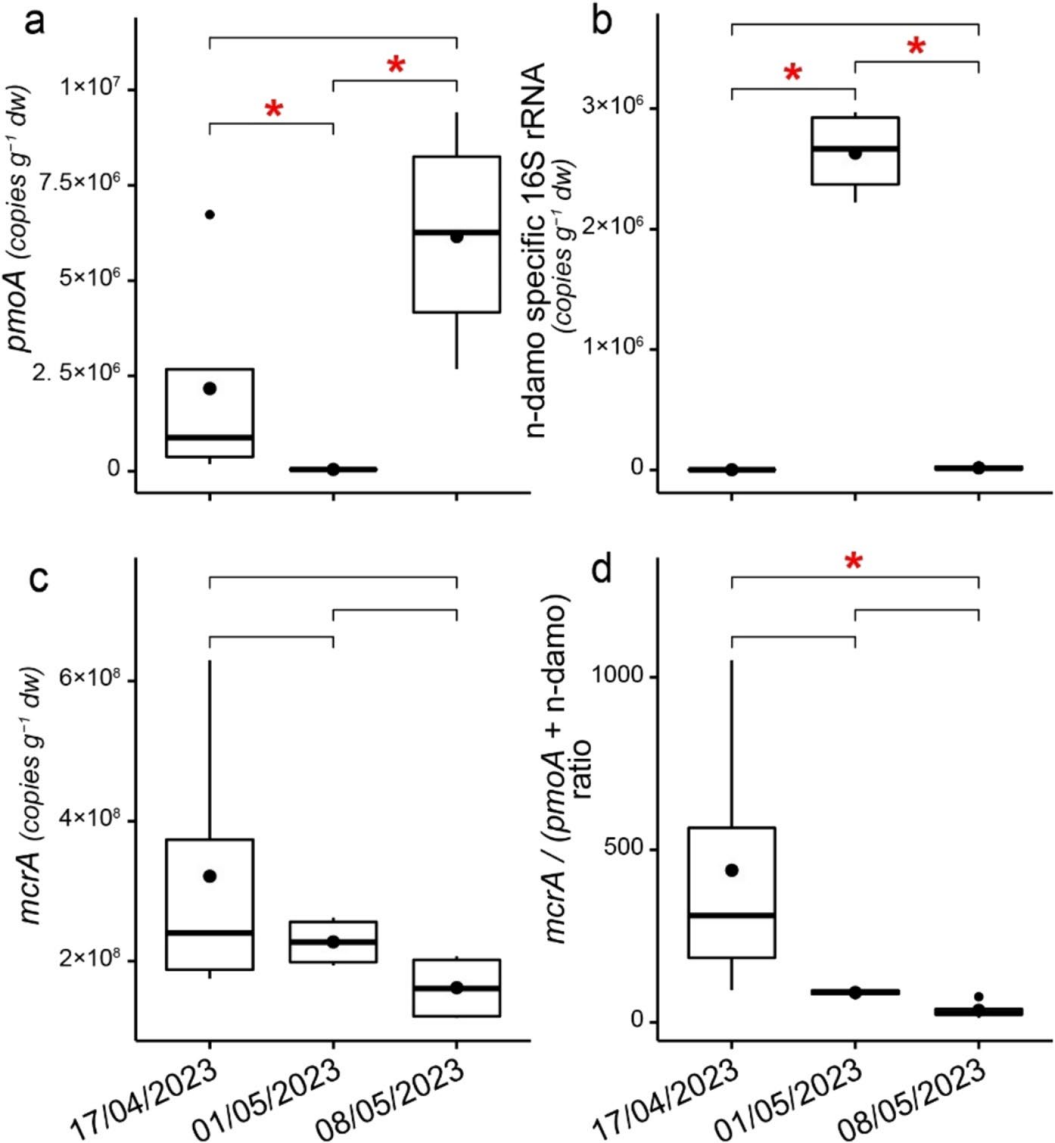
Boxplots of the CH_4_ cycle functional gene abundances (gene copies per gram of dry soil) of **(a)** *pmoA*, **(b)** n-damo specific 16S rRNA, **(c)** *mcrA*, and **(d)** the methanogenesis to methanotrophy (*mcrA* / (*pmoA* + n-damo specific 16S rRNA)) ratio on soil sampling dates (17/04/2023, 01/05/2023, 08/05/2023). Median values are marked with solid lines, mean values with circles, 25th and 75th percentiles with box boundaries and outliers with dots. Asterisks above bars show statistically significant differences between gene abundances on different days

The prokaryotic abundances and their proportions are presented in Supplementary Table S3. The temporal variation of gene abundances and ratios on different measurement days is shown in Fig. 5 for the N cycle genes and Fig. 6 for the CH_4_ cycle genes. The *nosZ* I and *nosZ* II gene abundances decreased during the sampling period, while archaeal *amoA*, COMAMMOX, *nirS* and *nirK* were highest in the middle of the sampling period. Bacterial *amoA* was highest towards the end of the sampling period. The *nir/amoA* ratio did not alter significantly during the study period, and *nir/nosZ* increased after the beginning but then remained stable (Fig. 5). Significant negative correlations occurred between stem and soil N_2_O fluxes and bacterial 16S rRNA abundance, as well as denitrifying *nosZ* I and *nosZ* II gene abundances (Fig. 3)*.* Significant positive relationships also occurred between soil N_2_O fluxes and the *nir* gene (*nirK* and *nirS*) to *nosZ* gene (*nosZ* I and *nosZ* II) ratio. Spruce N_2_O fluxes correlated negatively with the abundance of *nirK* genes and the *nir/amoA* gene ratio (Fig. 3).

The abundances and ratios of the methanogenic *mcrA* and methanotrophic *pmoA* and n-damo 16S rRNA genes are presented in Fig. 6 and Supplementary Table S3. While the *mcrA* gene abundance did not show significant temporal variation between soil sampling dates, the *pmoA* gene was more prevalent on the last sampling day, and n-damo abundance peaked in the middle of the measurement period (Fig. 6). Accordingly, the ratio of methanogenic genes to methanotrophic genes decreased through the measurement period (Fig. 6). Birch CH_4_ and CO_2_, and soil CO_2_ fluxes, were negatively correlated with bacterial and archaeal 16S rRNA gene abundances, while spruce CH_4_ fluxes were negatively correlated with bacterial 16S rRNA gene abundance. Birch, spruce and soil CH_4_ fluxes, as well as birch and soil CO_2_ fluxes, had significant positive correlations with n-damo specific gene abundance. Birch and spruce CH_4_ and soil CO_2_ fluxes correlated negatively with the ratio of methanogenic-to-methanotrophic genes (Fig. 2).

The PCA further shows that the principal components differed across the soil sampling dates, indicating that different functional genes dominate the variability of observations on different days for both the N and CH_4_ cycles (Supplementary Fig. S8).

## Discussion

Spring can be an overlooked period of the year for stem and soil GHG fluxes. Soil freeze–thaw events can be crucial hot moments of N_2_O (Kazmi et al. 2023; Mander et al. 2021) and continuous wet conditions after snowmelt can promote CH_4_ release (Ranniku et al. 2024). We observed low emissions from stems and soil that increased towards the end of April and beyond (Fig. 1). CH_4_ release from birch stems in peatland forests has been shown to stay low but detectable in the winter and increase in the spring following the rising water table after snowmelt (Pangala et al. 2015; Ranniku et al. 2024). Meanwhile, previous studies have demonstrated soil fluxes fluctuating around zero in the winter (Ranniku et al. 2023), with CH_4_ uptake prevailing during the onset of spring (Pihlatie et al. 2010). We did not detect soil CH_4_ uptake as observed in the same study area in spring of 2021 (Ranniku et al. 2023). Our observed forest floor CH_4_ release was more similar to previous measurements in temperate wetland forests, where emissions prevailed over uptake, increasing from winter to spring (Mander et al. 2022; Pangala et al. 2015). Similarly to previous measurements (Ranniku et al. 2024), birch stem CH_4_ fluxes exceeded spruce fluxes during the study period. Inter-species variations can be linked to microtopographical variations in water availability and soil water gas concentrations (Machacova et al. 2019; Terazawa et al. 2015), differences in tree root system depth and density (Bachofen et al. 2024; Puhe 2003), and differences in stem morphology, xylem structures, and bark characteristics between coniferous and broadleaved tree species (Jeffrey et al. 2023a; Salomón et al. 2016; Teskey et al. 2008). We observed stem and soil CH_4_ fluxes to be negatively related to SWC and WTD. In addition, a significant relationship between soil CH_4_ fluxes and WTD did not emerge, while it is generally considered to be one of the main drivers of soil CH_4_ dynamics (Ojanen et al. 2010) (Fig. 2). Thus, the first proposed hypothesis was not supported, as elevated water levels did not indicate higher CH_4_ emissions. As variations in WTD and SWC during the study period were relatively small (Fig. 1a), soil hydrological conditions may be more important for long-term CH_4_ flux dynamics, for example with changes between dry and wet periods, and are not direct governing factors in the short-term. For stem fluxes from birch trees, which have low-reaching rooting systems (Bachofen et al. 2024), this may be due to stem-emitted CH_4_ originating from deeper soil layers where methanogenesis prevails, as it is taken up by tree roots and moves up the xylem. In that case, WTD and SWC changes happening in the topsoil layers would have a less significant effect on stem CH_4_ emissions (Pangala et al. 2015). The temporal dynamics of CH_4_ fluxes from the soil were best explained by changes in temperature, while PAR was the primary driver of stem CH_4_ emissions. As PAR is closely linked to plant physiological activity, it may serve as a proxy for sap flow, which facilitates the upward transport of water and dissolved gases in the stem (Bovard et al. 2005). While sap flow was not measured in this study, the significant relationship between PAR and stem CH_4_ flux suggests that sap flow may already influence gas transport early in the growing season, before or near bud burst. Furthermore, PAR influence could be linked to aerobic, abiotic methanogenesis in plants (Covey & Megonigal 2019; Keppler et al. 2006), triggered by ultraviolet radiation (Tenhovirta et al. 2022; Vigano et al. 2008). However, *in-situ* evidence of the process, as well as specific observations from tree stems, are lacking. The different drivers of stem and soil fluxes, together with the non-significant correlation between the two, could indicate decoupling of stem and soil CH_4_ fluxes (Barba et al. 2024; Pitz & Megonigal 2017).

The highest N_2_O fluxes observed here were much lower in comparison to those previously reported during the spring at the same site, where soil hydrological conditions showed comparatively more variation (Ranniku et al. 2023). Previous studies have demonstrated that N_2_O emissions during winter and spring can be substantial contributors to the net annual N_2_O release from the soil (Mander et al. 2021; Viru et al. 2020), as well as tree stems (Mander et al. 2021; Ranniku et al. 2023), driven by hot moments of emissions. However, the exact timing of these springtime peaks has varied and remains an uncertainty due to inadequate measurement frequencies (Groffman et al. 2006; Pihlatie et al. 2010). In the multi-year study in a riparian alder forest, springtime peaks occurred during the freeze–thaw period in February–March 2018. In a previous study in the same study area, stem N_2_O emissions increased in mid-March, while peaks of different magnitude occurred for soil emissions in January, March and April (Ranniku et al. 2023). The relatively stable soil hydrological conditions observed during the current study period support previous findings that soil and stem N_2_O emissions, and their hot moments, are triggered by more rapid changes in soil hydrologic conditions (Mander et al. 2021; Ranniku et al. 2024). The lack of substantial temporal variations in SWC and a consistent lowering of the WTD further indicate that the freeze–thaw period with potentially greater fluctuations in soil hydrological conditions occurred prior to our measurement period. However, if SWC was in optimum range for N_2_O production, and thus not a limiting factor for N_2_O production and release, temperature showed the strongest correlation with fluxes. PAR was also significantly linked to N_2_O fluxes from birch stems, likely due to indirect effects through temperature and showing the influence of plant physiological activity on stem N_2_O release (Fig. 3).

Similarly to CH_4_ and N_2_O, temporal dynamics of CO_2_ fluxes followed the trend of temperature, peaking on 25/04/2023, decreasing slightly and increasing again in May. Soil and stem CO_2_ efflux has been shown to relate to the growing season, influenced by the temperature sensitivity of plant respiration and diffusion rates (Teskey et al. 2008). In agreement with previous studies, T_soil_, T_air_ and PAR have the strongest influence of stem CO_2_ efflux. Higher temperature leads to higher respiration rates, while PAR has an effect on tree photosynthesis, influencing tree growth (Zha 2004). The negative correlations observed between CO_2_ fluxes and soil water parameters support higher respiration rates and increased organic matter decomposition due to enhanced microbial activity under elevated oxygen availability (Jiang et al. 2020; Schindlbacher et al. 2009). Thus, the temporal dynamics of fluxes of all gases showed evidence of being related to plant phenological and physiological activity during the very early stages of the growing season.

Our study presented one of the first attempts to explore the connection between birch stem CH_4_ and CO_2_ fluxes and the dissolved gas concentrations in birch sap and soil water. While prior research has highlighted the significance of soil porewater CH_4_ concentration in regulating stem CH_4_ release in temperate and tropical wetland forests (Pangala et al. 2015, 2013), our focus on sap concentrations offers a novel perspective. Temporal patterns of dCH_4soil_ and dCO_2soil_ showed that increased gas concentrations in soil water precede a peak in stem and soil fluxes, as well as elevated dCH_4sap_ and dCO_2sap_. This indicates that there is a delay between increased gas concentrations in soil water and emissions from soil and stems. However, no significant correlations were observed between soil water gas concentrations and fluxes, making it difficult to establish a clear direct relationship.

No clear evidence was observed to support the second hypothesis of stem CH_4_ emissions being directly related to birch sap gas concentrations. The temporal patterns of birch CH_4_ fluxes did not align with those of dCH_4sap_, as peak fluxes occurred before the highest values of dCH_4sap_. In addition, no significant correlation was observed between birch CH_4_ fluxes and dCH_4sap_ at any measured stem height, suggesting that emissions may not be directly linked to the transpiration stream during this early growing season. However, the significant relationship between PAR and stem CH_4_ flux, along with the decreasing CH_4_ flux trend with stem height, suggests that stem emissions may still be soil-derived. This process, however, may be decoupled from sap gas concentrations, pointing to alternative transport mechanisms. For instance, Jeffrey et al. (2023a) proposed that trees with layered bark structures have potential for axial bark-mediated gas diffusion, occurring independently of sap flow—a mechanism that could also apply to birch trees.

We found that dCH_4soil_ was approximately 40 times higher than dCH_4sap_, while dCO_2soil_ and dCO_2sap_ remained similar. This may be due to methanotrophs within the stem oxidising some of the CH_4_ during its ascent in the soil-stem transpiration stream (Putkinen et al. 2021). Additionally, significant correlations emerged between CO_2_ fluxes and dCO_2sap_, with simultaneous increases until reaching peak values on 25/04/2023. As stem respiration also produces CO_2_, which can be diffused out into the atmosphere but also further dissolve in the xylem sap, the interpretation of dCO_2sap_ remains challenging (Hölttä & Kolari 2009; Teskey et al. 2008). Furthermore, while diurnal variations of CO_2_ concentrations in xylem sap have been previously reported (McGuire & Teskey 2004; Saveyn et al. 2007), CH_4_ remains largely unstudied. Water ascent from tree roots through the xylem can occur within hours, potentially revealing shorter-term time lags between soil and stem dissolved gas concentrations and gas fluxes (Schenk et al. 2016). Therefore, further investigation into diurnal variations in stem fluxes in relation to sap flow rates and dissolved gas concentrations would provide valuable insights into plant hydraulics and its effect on fluxes. In addition, isotopic studies could help clarify the depth of root water uptake belowground. Recently, increasing valuable efforts have been made to explore the effects of sap flow (Anttila et al. 2023), plant hydraulics (Megonigal et al. 2020), and tree stem physiological traits (Jeffrey et al. 2023a) on stem GHG fluxes, and the use of labelled ^13^CH_4_ in deep soil layers to understand transport processes (Plain and Epron 2021). However, integrating sap flow and isotopic approaches to study gas transport mechanisms remains an underexplored and promising area of research.

The integration of stem and soil GHG flux dynamics and the associated soil chemistry and microbiology remain scarce in existing research. Although the underlying soil chemistry and microbial community composition may not directly drive tree stem GHG fluxes, they can have an indirect effect by regulating soil GHG production and consumption processes and the subsequent gas uptake by tree roots. Therefore, strong relationships between soil chemical and microbiological parameters and stem fluxes could give further indication of the soil origin of stem-emitted GHGs.

Changes in soil hydrologic condition, availability of labile substrate, and microbial properties in the spring, following the freeze–thaw periods, jointly affect the production and release of GHGs from the soils. Agreeing with the third hypothesis, we observed significant changes in the abundances of functional genes between measurement days, with different genes driving the variability of observations across days for both the N and CH_4_ cycles. During the study period, soil NO_3_ content displayed similar temporal trends to GHG flux dynamics, peaking at the end of April, followed by consumption. NO_3_ production has been shown to remain low in the winter, as nitrifiers are inhibited by cold temperatures (Masta et al. 2023; Smith et al. 2010). Meanwhile, NH_4_ content was highest at the beginning of the study period and was consumed in the topsoil as spring progressed. Previous studies have shown large NH_4_ pools in the winter (Ueda et al. 2015), due to soil freezing, which increases labile substrate availability from the die-off of microbes and fine roots (Groffman et al. 2006). NH_4_ content decreased alongside with WTD and SWC, suggesting that nitrification occurs in the soil under increasingly aerobic conditions as indicated by rising NO_3_ content, a product of nitrification. The positive relationships between soil NO_3_ content and stem fluxes, as well as soil CO_2_ efflux, were likely also indirect, affected by temperature dynamics. On the other hand, we also observed genetic potential for complete denitrification at the beginning of the study period, indicated by the low *nir/nosZ* gene ratio, under high WTD and SWC conditions at low temperatures. Thus, potential for both nitrification and complete denitrification were observed, resulting in low soil N_2_O emissions at the beginning of the study period. In the middle of the measurement period, NO_3_ levels declined, likely due to conversion to N_2_O through incomplete denitrification, indicated by high *nirS* and *nirK* gene abundances, a high* nir/nosZ* ratio, and elevated soil N_2_O release compared to the beginning of the study period. It is likely that our weekly soil sampling frequency was insufficient to capture the true peak of NO_3_ in the soil and the 25/04/2023 peak already reflected the reduced availability of NO_3_ for denitrification, while N_2_O was being produced and emitted. Thereafter, the NO_3_ pool was depleted and N_2_O emissions decreased.

We observed a gradual decrease in methanogenic potential and increase in methanotrophic potential, indicated by the declining methanogenic-to-methanotrophic gene ratio over the measurement period. Previous studies in forest soils have shown that higher rates of N cycling, induced by soil thawing and wetting, inhibit CH_4_ uptake (Wu et al. 2020). On the second measurement day, the n-damo specific genes were driving the methanotrophic abundance, which switched to *pmoA* gene dominance by the end of the study. The gene ratio *mcrA*/(*pmoA* + n-damo) negatively correlated with soil CO_2_ fluxes, possibly to a small extent due to CH_4_ oxidation to CO_2_ (Hu et al. 2014). However, only genes related to the n-damo process correlated with soil CH_4_ fluxes. This correlation was positive, although n-damo is typically responsible for CH_4_ consumption in the aerobic-anaerobic interface under high levels of nitrate (Hu et al. 2014; Zhou et al. 2014). Additionally, abundance of the methanotrophic *pmoA* genes was higher at the end of the study, coinciding with lower emissions. However, fluxes did not significantly correlate with *pmoA* abundance. CH_4_ emissions from birch stems correlated with soil NH_4_ and NO_3_ levels, with more CH_4_ being emitted when NH_4_ content in the topsoil was low. This was likely due to NH_4_ conversion to NO_3_ under warmer and drier conditions, coinciding with higher emissions. Under aerobic topsoil conditions, N availability has been shown to enhance the growth and activity of methanotrophs, further decreasing net CH_4_ emission or increasing CH_4_ uptake (Aronson & Helliker 2010; Bodelier & Laanbroek 2004). The negative correlation between stem CH_4_ fluxes and the ratio of methanogenic-to-methanotrophic genes could indicate a decoupling of stem CH_4_ emissions from soil processes occurring in the topsoil. These results highlight the need for a more detailed analysis of the relationships between the CH_4_-cycle genes and fluxes under different environmental conditions (Espenberg et al. 2024).

## Conclusion

To conclude, during spring in a drained peatland forest, initial low emissions of CH_4_, N_2_O, and CO_2_ from stems and soil increased towards late April. The stable soil hydrologic conditions, optimal for CH_4_ and N_2_O production, had minimal short-term impact on fluxes, with temperature primarily driving soil and stem fluxes, alongside PAR influencing stem fluxes. No clear evidence linked stem CH_4_ emissions to birch sap gas concentrations, while interpretation of the evident relationship between CO_2_ emissions and birch sap gas concentrations remains a challenge. We observed shifts in soil microbial N and CH_4_-cycling community composition throughout the spring. Potential for methanogenesis and complete denitrification was higher under elevated SWC, shifting to methanotrophy and incomplete denitrification as the study progressed. However, our results underscore the need for further analysis of microbial CH_4_ and N cycles and their impact on GHG fluxes under varying environmental conditions. Soil sampling was performed at the top 10 cm, which may not reflect the soil conditions at which tree roots absorb water, especially after WTD falls below 10 cm, reducing the circulation of nutrients in soil water. Thus, determining the depth of the root profile of different tree species, along with water uptake depth, and soil sampling at various depths would be important next steps to elucidate the related processes.

## Supplementary Information

Below is the link to the electronic supplementary material.

Supplementary file1 (DOCX 2500 kb)

## Data Availability

The data that support the findings of this study are available from the corresponding author upon reasonable request.
