# Supplementary material for: Springtime soil and tree stem greenhouse gas fluxes and the related soil microbiome pattern in a drained peatland forest
Source: Biogeochemistry. 2025 May 7;168(3):48. doi: 10.1007/s10533-025-01238-3 (PMC12058906; doi:10.1007/s10533-025-01238-3)
Supplement: Supplementary file 1 — Supplementary file1 (DOCX 2500 kb) [file 10533_2025_1238_MOESM1_ESM.docx]

**Springtime soil and tree stem greenhouse gas fluxes and the related soil microbiome pattern in a drained peatland forest**

Reti Ranniku^1^*, Fahad Ali Kazmi^1^, Mikk Espenberg^1^, Joosep Truupõld^1^, Jordi Escuer-Gatius^2^, Ülo Mander^1^, Kaido Soosaar^1^

^1^ Department of Geography, Institute of Ecology & Earth Sciences, University of Tartu, 46 Vanemuise, EST-51014 Tartu, Estonia

^2^ Institute of Agricultural and Environmental Sciences, Estonian University of Life Sciences, 5 Fr.R. Kreutzwaldi, EST-51006 Tartu, Estonia

* Corresponding author: Reti Ranniku, [reti.ranniku@ut.ee](mailto:reti.ranniku@ut.ee)

**Supplementary Information**

This Supplementary Information (SI) provides additional data and detailed analyses on greenhouse gas (GHG) fluxes and their environmental drivers. It includes details on the study site (Tables S1, S2) and experimental design (Fig. S1), as well as the measurement set-up for tree stem chamber systems (Fig. S2). The SI also provides more detailed data on gene abundances in the studied soils (Table S3), as well as mixed-effects model results for GHG flux predictors (Table S4). The SI also features figures illustrating continuous automated time series measurements of soil GHG fluxes (Fig. S3), vertical profiles of birch and spruce GHG fluxes at different heights (Fig. S4), regression analyses linking GHG fluxes with air temperature (T_air_), photosynthetically active radiation (PAR) (Fig. S5), and dissolved gas concentrations in birch sap and soil water (Fig. S6), temporal dynamics of soil NO_3_ and NH_4_ contents (Fig. S7), and Principal Component Analysis (PCA) of soil, birch, and spruce GHG fluxes, environmental variables, and gene abundances (Fig. S8). This SI complements the main text by providing deeper insights into flux variability, environmental interactions, and methodological details.

**Supplementary Table S1** Soil chemical properties of the study site (mean ± standard error, n=12). Soil samples collected from 0-10 cm soil depth.

| Date | NO_3_^−^−N  (mg/kg) | NH_4_^+^−N  (mg/kg) | Inorganic N  (mg/kg) | TC  (%) | TN  (%) | C:N | pH |
| --- | --- | --- | --- | --- | --- | --- | --- |
| 17/04/2023 | 4.99 ± 1.02 | 16.3 ± 3.1 | 21.3 ± 2.8 | 39.7 ± 1.1 | 2.60 ± 0.06 | 15.3 ± 0.2 | 4.69 ± 0.09 |
| 25/04/2023 | 17.5 ± 3.4 | 7.33 ± 2.40 | 24.9 ± 4.1 |  |  |  |  |
| 01/05/2023 | 6.59 ± 1.61 | 5.02 ± 1.22 | 11.6 ± 1.1 |  |  |  |  |
| 08/05/2023 | 6.30 ± 1.43 | 8.64 ± 2.53 | 14.9 ± 1.6 | 39.7 ± 0.6 | 2.66 ± 0.05 | 15.0 ± 0.2 | 4.72 ± 0.05 |

**Supplementary Table S2** Tree stand characteristics. Based on Becker et al. (2018).

| Tree species | Number of trees (# ha^-1^) | Average stand height (m) | Average stem diameter at breast height 1.3 m (cm) | Basal area (m^2^ ha^−1^) | Tree biomass stock (m^3^ ha^−1^) |
| --- | --- | --- | --- | --- | --- |
| Downy birch | 1660 | 15 | 14.0 | 24.5 | 193 |
| Norway spruce | 942 | 17 | 18.4 | 25 | 208 |

**Supplementary Table S3** Average values (n=12) and standard errors of gene parameter values in the studied soils.

|  | | Date | Abundance (gene copies per g^−1^ dry weight) | Proportion in total prokaryotic abundance  (%) |
| --- | --- | --- | --- | --- |
| Prokaryotes | Bacterial 16S rRNA | 17/04/2023 | 1.96 × 10^11^ ± 9.15 × 10^9^ | 98.6 ± 0.3 |
|  |  | 01/05/2023 | 1.74 × 10^11^ ± 4.57 × 10^9^ | 99.0 ± 0.2 |
|  |  | 08/05/2023 | 1.51 × 10^11^ ± 1.15 × 10^10^ | 98.9 ± 0.2 |
|  | Archaeal 16S rRNA | 17/04/2023 | 2.62 × 10^9^ ± 5.26 × 10^8^ | 1.36 ± 0.31 |
|  |  | 01/05/2023 | 1.80 × 10^9^ ± 4.12 × 10^8^ | 1.02 ± 0.22 |
|  |  | 08/05/2023 | 1.63 × 10^9^ ± 3.14 × 10^8^ | 1.09 ± 0.18 |
| Nitrifiers | Archaeal *amoA* | 17/04/2023 | 2.11 × 10^7^ ± 4.98 × 10^6^ | 0.010 ± 0.002 |
|  |  | 01/05/2023 | 9.04 × 10^7^ ± 1.56 × 10^7^ | 0.051 ± 0.009 |
|  |  | 08/05/2023 | 4.38 × 10^6^ ± 1.67 × 10^6^ | 0.003 ± 0.001 |
|  | Bacterial *amoA* | 17/04/2023 | 3.06 × 10^7^ ± 6.15 × 10^6^ | 0.015 ± 0.003 |
|  |  | 01/05/2023 | 1.53 × 10^7^ ± 2.85 × 10^6^ | 0.009 ± 0.002 |
|  |  | 08/05/2023 | 3.87 × 10^7^ ± 7.1 × 10^6^ | 0.025 ± 0.004 |
|  | COMAMMOX *amoA* | 17/04/2023 | 2.84 × 10^6^ ± 5.88 × 10^5^ | 1.40 × 10*^–^*^3^ ± 2.27 × 10*^–^*^4^ |
|  |  | 01/05/2023 | 5.80 × 10^5^ ± 9.01 × 10^4^ | 3.33 × 10*^–^*^4^ ± 5.31 × 10*^–^*^5^ |
|  |  | 08/05/2023 | 2.50 × 10^6^ ± 2.47 × 10^5^ | 1.68 × 10*^–^*^3^ ± 2.27 × 10*^–^*^4^ |
| Denitrifiers  (Nitrite reductase) | *nirS* | 17/04/2023 | 3.16 × 10^7^ ± 4.06 × 10^6^ | 0.016 ± 0.002 |
|  |  | 01/05/2023 | 7.57 × 10^7^ ± 9.52 × 10^6^ | 0.042 ± 0.005 |
|  |  | 08/05/2023 | 2.57 × 10^7^ ± 1.64 × 10^6^ | 0.018 ± 0.002 |
|  | *nirK* | 17/04/2023 | 1.10 × 10^9^ ± 1.37 × 10^8^ | 0.557 ± 0.062 |
|  |  | 01/05/2023 | 2.93 × 10^9^ ± 3.18 × 10^8^ | 1.70 ± 0.21 |
|  |  | 08/05/2023 | 6.38 × 10^8^ ± 1.06 × 10^8^ | 0.417 ± 0.053 |
| Denitrifiers  (Nitrous oxide reductase) | *nosZI* | 17/04/2023 | 6.50 × 10^8^ ± 6.07 × 10^7^ | 0.329 ± 0.030 |
|  |  | 01/05/2023 | 5.50 × 10^8^ ± 4.13 × 10^7^ | 0.313 ± 0.022 |
|  |  | 08/05/2023 | 1.29 × 10^8^ ± 1.17 × 10^7^ | 0.088 ± 0.008 |
|  | *nosZ*II | 17/04/2023 | 2.19 × 10^9^ ± 1.71 × 10^8^ | 1.107 ± 0.081 |
|  |  | 01/05/2023 | 8.12 × 10^8^ ± 8.68 × 10^7^ | 0.465 ± 0.052 |
|  |  | 08/05/2023 | 2.35 × 10^8^ ± 3.15 × 10^7^ | 0.152 ± 0.014 |
| Methanogenesis | *mcrA* | 17/04/2023 | 2.52 × 10^8^ ± 3.72 × 10^7^ | 0.132 ± 0.022 |
|  |  | 01/05/2023 | 2.43× 10^8^ ± 1.49 × 10^7^ | 0.140 ± 0.010 |
|  |  | 08/05/2023 | 2.13 × 10^8^ ± 3.62 × 10^7^ | 0.147 ± 0.023 |
| Methanotrophy | *pmoA* | 17/04/2023 | 1.10 × 10^6^ ± 5.22 × 10^5^ | 5.86 × 10*^–^*^4^ ± 2.94 × 10*^–^*^4^ |
|  |  | 01/05/2023 | 4.50 × 10^4^ ± 7.82 × 10^3^ | 2.61 × 10*^–^*^5^ ± 4.67 × 10*^–^*^6^ |
|  |  | 08/05/2023 | 6.05 × 10^6^ ± 7.85 × 10^5^ | 4.39 × 10*^–^*^3^ ± 7.05 × 10*^–^*^4^ |
|  | n-damo specific 16S rRNA | 17/04/2023 | 2.21 × 10^3^ ± 2.69 × 10^2^ | 1.13 × 10*^–^*^6^ ± 1.37 × 10*^–^*^7^ |
|  |  | 01/05/2023 | 2.12 × 10^6^ ± 1.89 × 10^5^ | 1.22 × 10*^–^*^3^ ± 1.15 × 10*^–^*^4^ |
|  |  | 08/05/2023 | 1.42 × 10^4^ ± 2.94 × 10^3^ | 9.07 × 10*^–^*^6^ ± 1.69 × 10*^–^*^6^ |

**Supplementary Table S4** Mixed-effects model results of model predictors (T_soil_, SWC, WTD) on birch, spruce and soil CH_4_, CO_2_, and N_2_O fluxes. Significant *p*-values are highlighted in bold.

|  |  | Predictor | T_soil_ | SWC | WTD |
| --- | --- | --- | --- | --- | --- |
| CH_4_ | Birch | Estimate | 0.05 | -3.737 | -0.109 |
|  |  | *p*-value | 0.725 | 0.358 | **0.029** |
|  | Spruce | Estimate | -0.012 | 0.166 | -0.059 |
|  |  | *p*-value | 0.810 | 0.756 | **0.002** |
|  | Soil | Estimate | 2.295 | -3.636 | 0.212 |
|  |  | *p*-value | **0.026** | 0.710 | 0.518 |
| N_2_O | Birch | Estimate | 0.264 | -0.358 | -0.038 |
|  |  | *p*-value | **0.004** | 0.777 | 0.199 |
|  | Spruce | Estimate | 0.206 | -0.119 | 0.076 |
|  |  | *p*-value | 0.335 | 0.958 | 0.319 |
|  | Soil | Estimate | 2.906 | 3.523 | -1.838 |
|  |  | *p*-value | **< 0.001** | 0.520 | **< 0.001** |
| CO_2_ | Birch | Estimate | 5153.9 | -17818.4 | -149.2 |
|  |  | *p*-value | **< 0.001** | 0.182 | 0.555 |
|  | Spruce | Estimate | 7163.2 | 23828.2 | 160.2 |
|  |  | *p*-value | **< 0.001** | 0.095 | 0.791 |
|  | Soil | Estimate | 7364.8 | 11902.4 | -3347.3 |
|  |  | *p*-value | **< 0.001** | 0.216 | **< 0.001** |


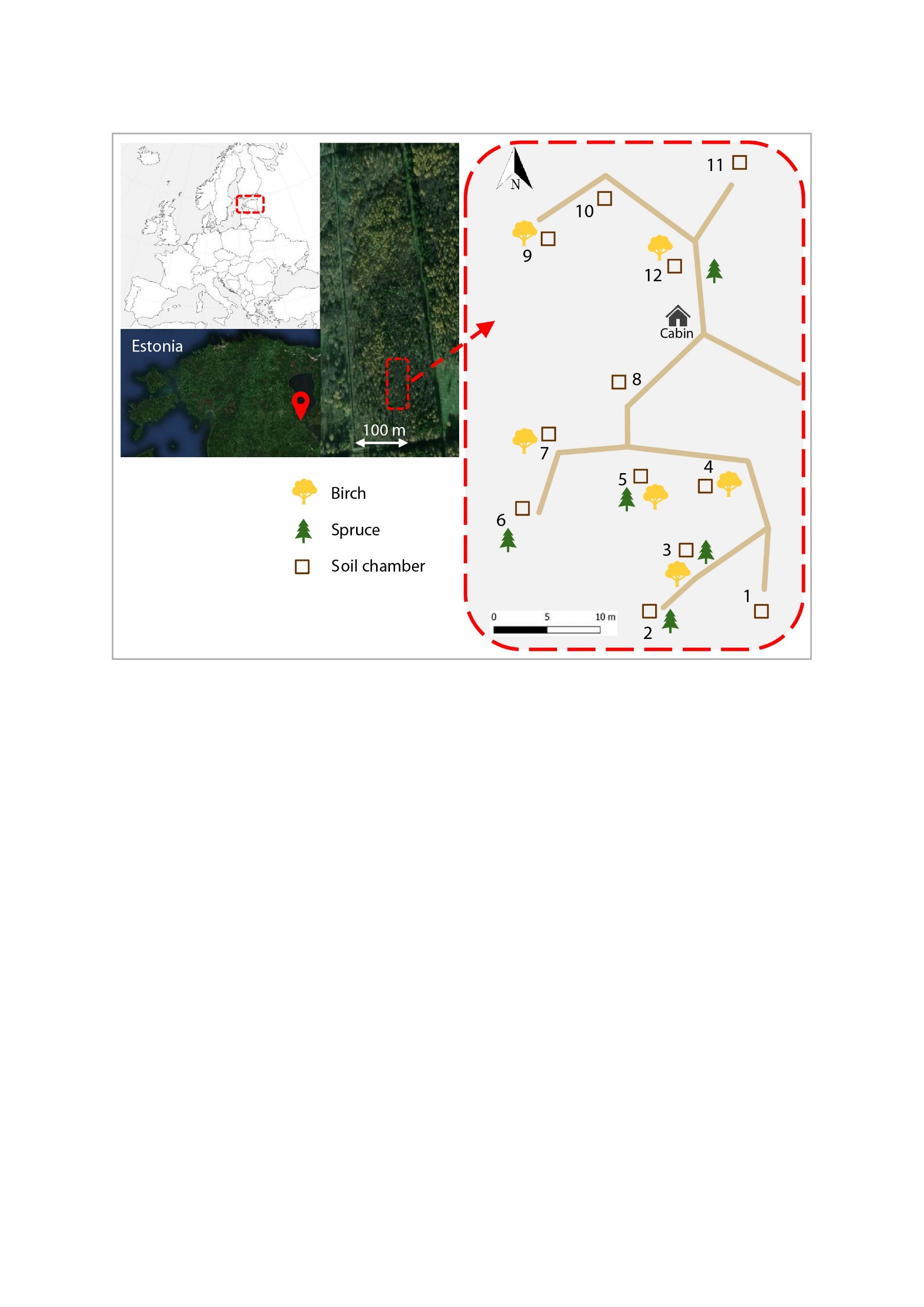


**Supplementary Fig.** **S1** Location of the study area and schematic view of the study site, including numbered monitoring points with soil chambers, birch, and spruce trees


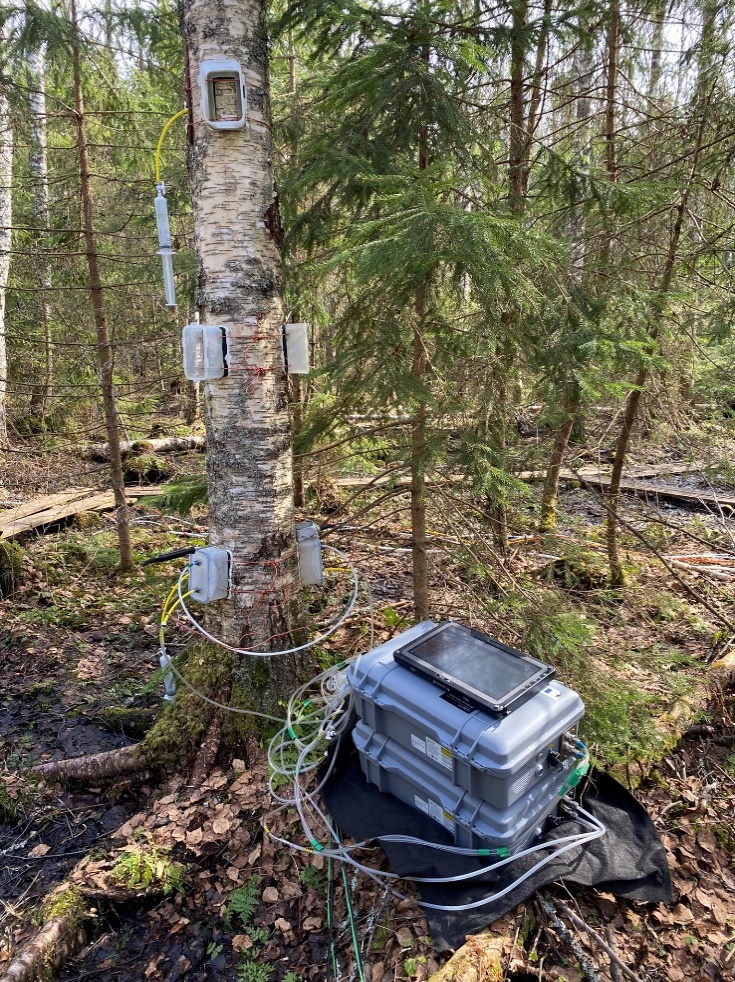


**Supplementary Fig. S2** Photo illustrating the measurement set-up of stem chamber systems on three heights connected to Li-Cor gas analysers, and birch sap collection. Photo credit: Reti Ranniku

**
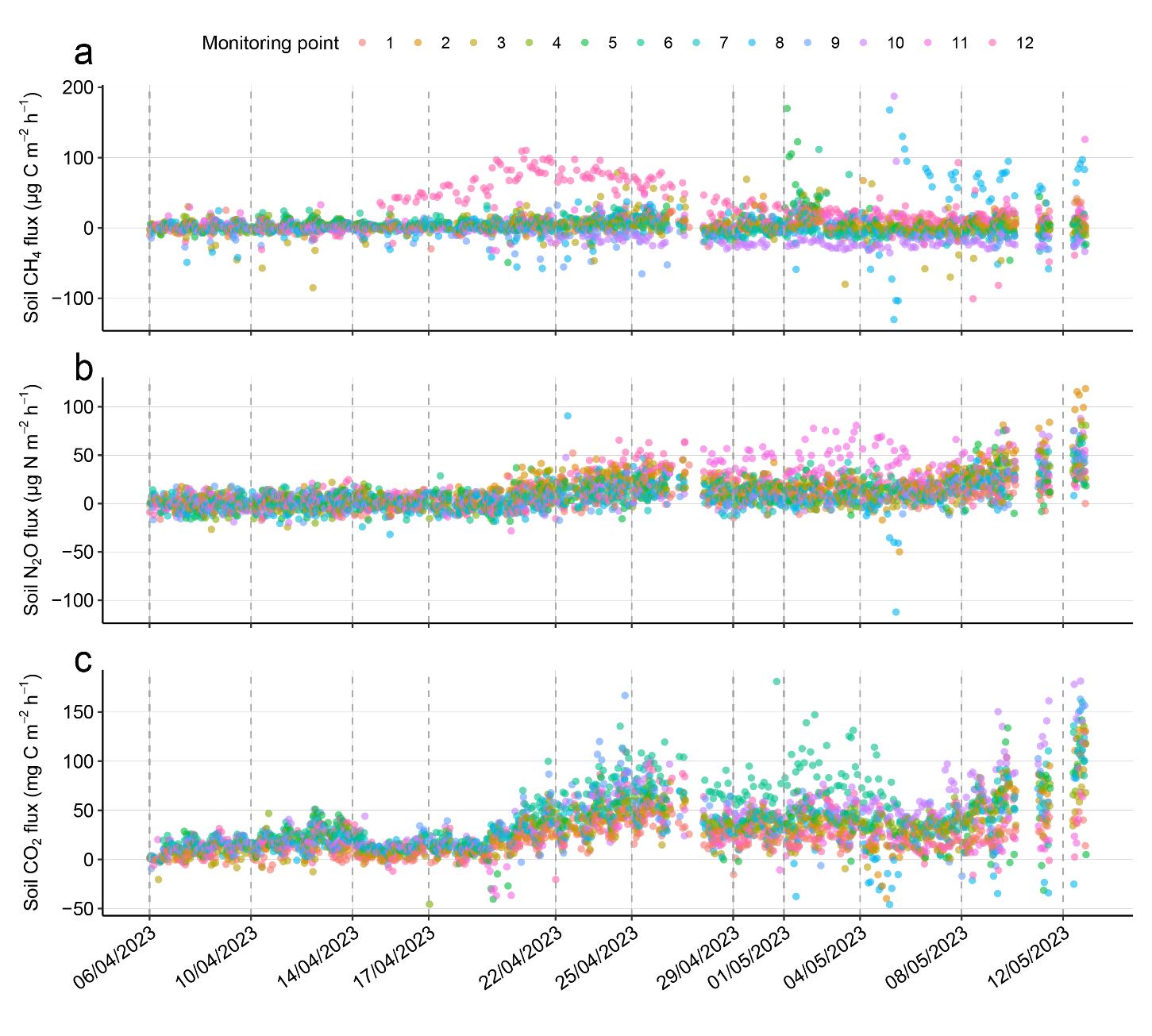
Supplementary Fig. S3** Continuous time series measurements of soil **(a)** CH_4_, **(b)** N_2_O, and **(c)** CO_2_ fluxes during the study period. Each point represents one automated chamber flux measurement. Measurements from different monitoring points have been displayed in different colours. The grey vertical dashed lines and the dates noted on the x-axis indicate specific dates where tree stem flux measurements were conducted (line at 00:00 on each date)


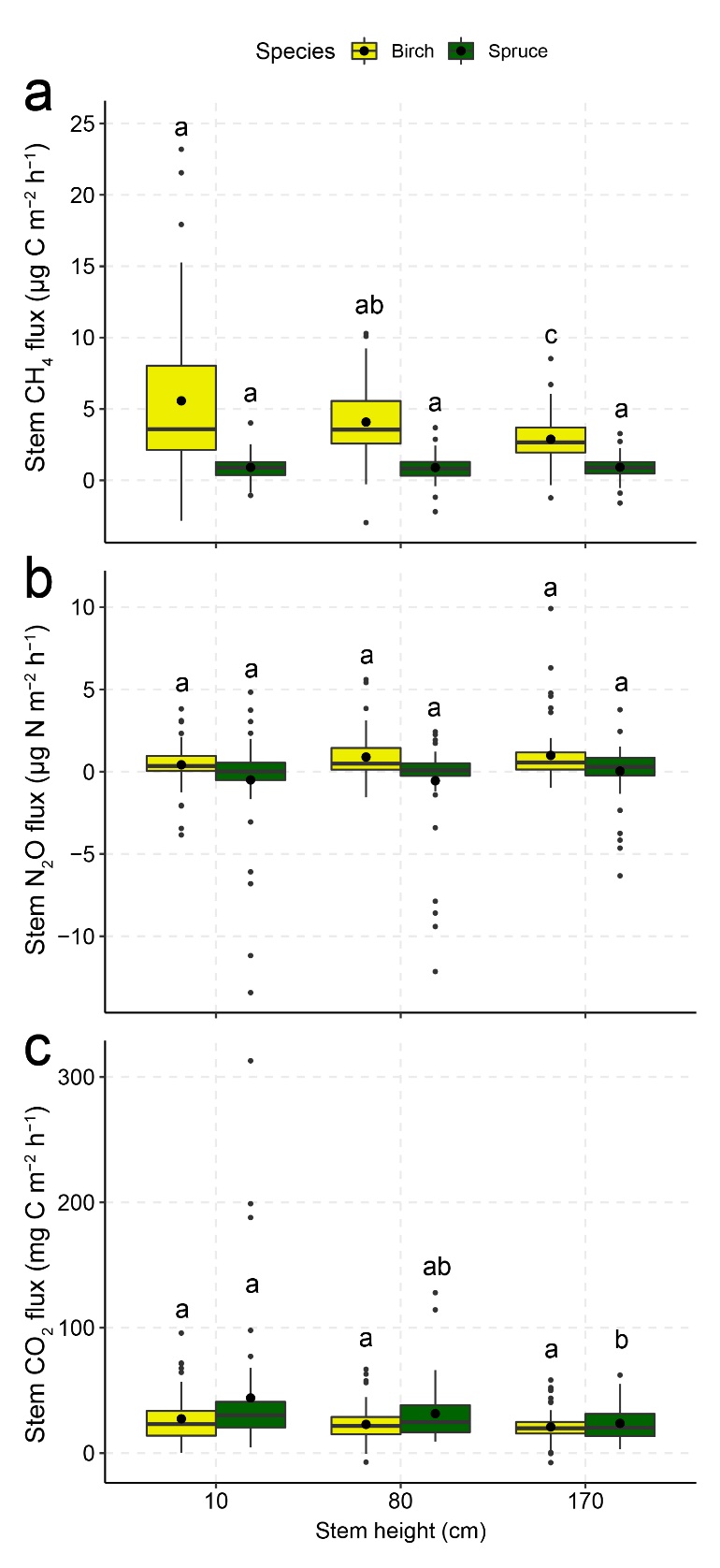


**Supplementary Fig. S4** Vertical profile of birch and spruce **(a)** CH_4_, **(b)** N_2_O and **(c)** CO_2_ fluxes at 10 cm, 80 cm and 170 cm, averaged across all plots and throughout the study period. Different letters above bars indicate statistically significant differences between fluxes at different heights within species, according to a Kruskal-Wallis one-way analysis of variance followed by a post-hoc Dunn test (*p* < 0.05). The boxes contain data within the 25th and 75th percentiles, the solid line within each box marks the median value, circles inside the boxes mark the mean values, and circles outside the boxes mark outliers


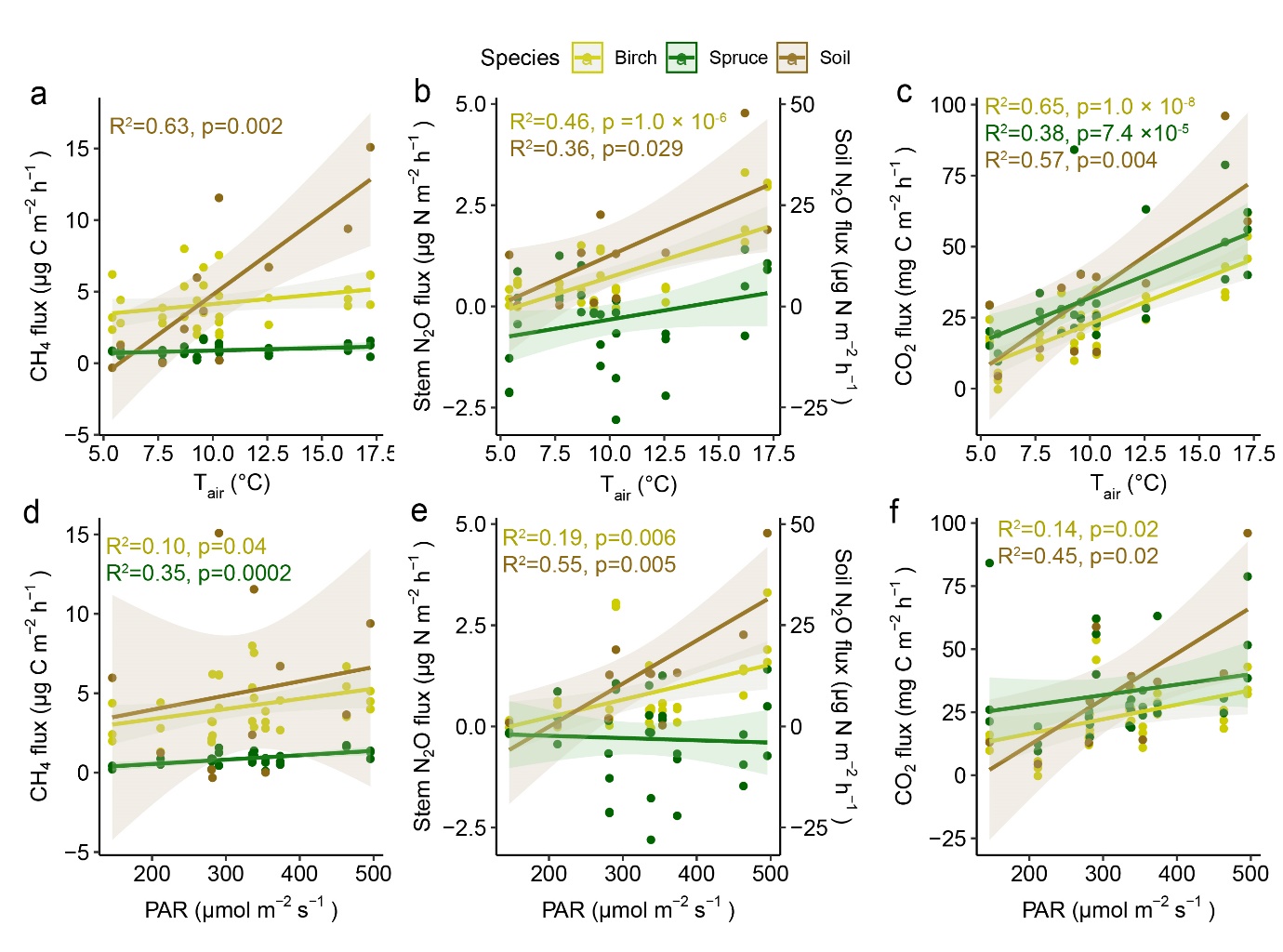
 **Supplementary Fig. S5** Relationship between T_air_ and birch, spruce, and soil **(a)** CH_4_, **(b)** N_2_O and **(c)** CO_2_ fluxes, and between PAR and birch, spruce, and soil **(d)** CH_4_, **(e)** N_2_O and **(f)** CO_2_ fluxes. Adjusted R^2^ and *p*-values of the relationships have been calculated according to the linear regression model. Only significant *p*-values have been shown


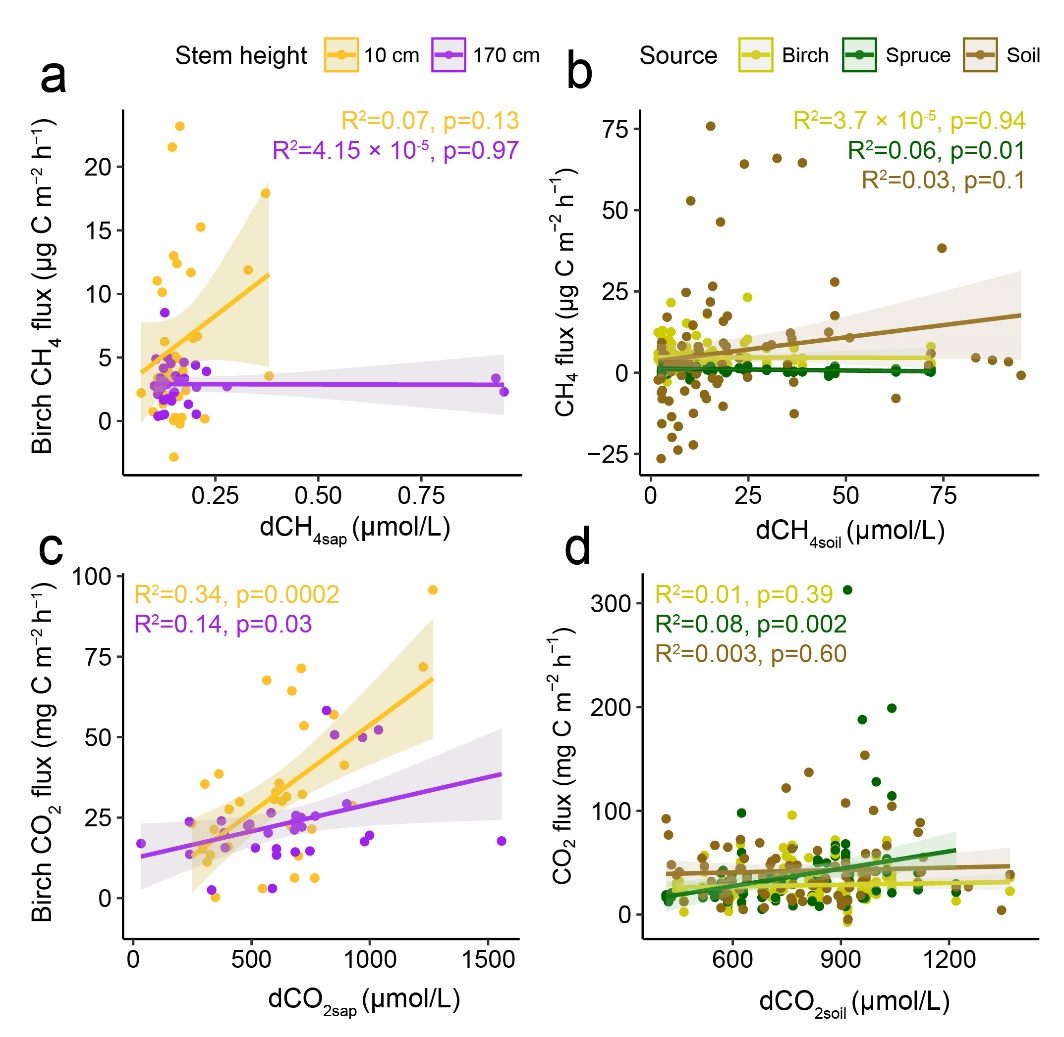


**Supplementary Fig. S6** Relationship between **(a)** birch stem CH_4_ flux and dissolved CH_4_ concentration in birch sap (dCH_4sap_) at 10 cm and 170 cm stem height; **(b)** soil and stem CH_4_ fluxes and dissolved CH_4_ concentrations in soil water (dCH_4soil_); **(c)** birch stem CO_2_ flux and dissolved CO_2_ concentration in birch sap (dCO_2sap_) at 10 cm and 170 cm stem height; **(d)** soil and stem CO_2_ fluxes and dissolved CO_2_ concentrations in soil water (dCO_2soil_) during birch sap sample collection dates (14/04/2023–04/05/2023) and soil water sample collection dates (17/04/2023–08/05/2023). Adjusted R^2^ and *p*-values of the relationships have been calculated according to the linear regression model


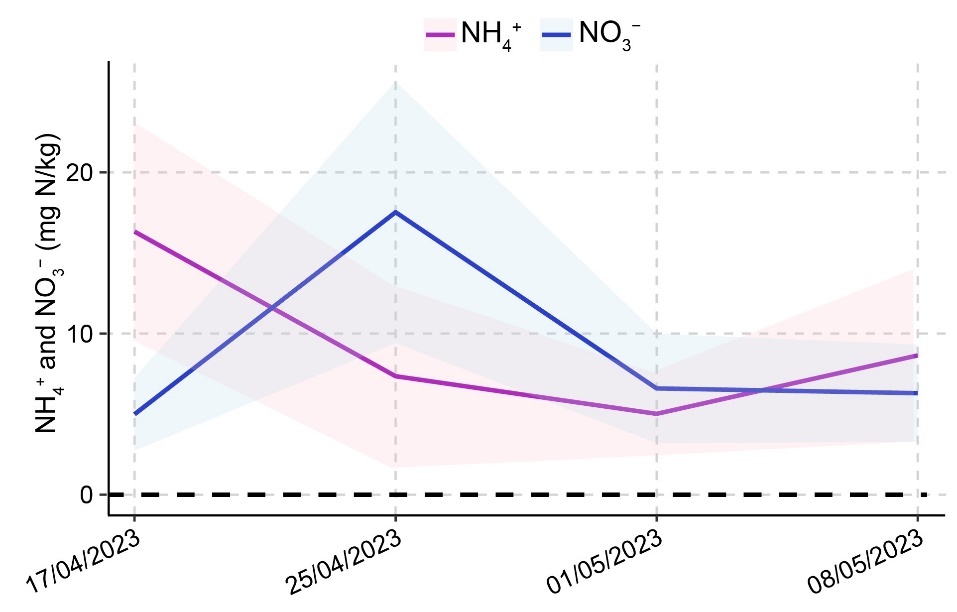


**Supplementary Fig. S7** Temporal dynamics of daily mean soil NO_3_ and NH_4_ contents (mg/kg) of the topsoil (0–10 cm) with 95% confidence intervals as the shaded area

**
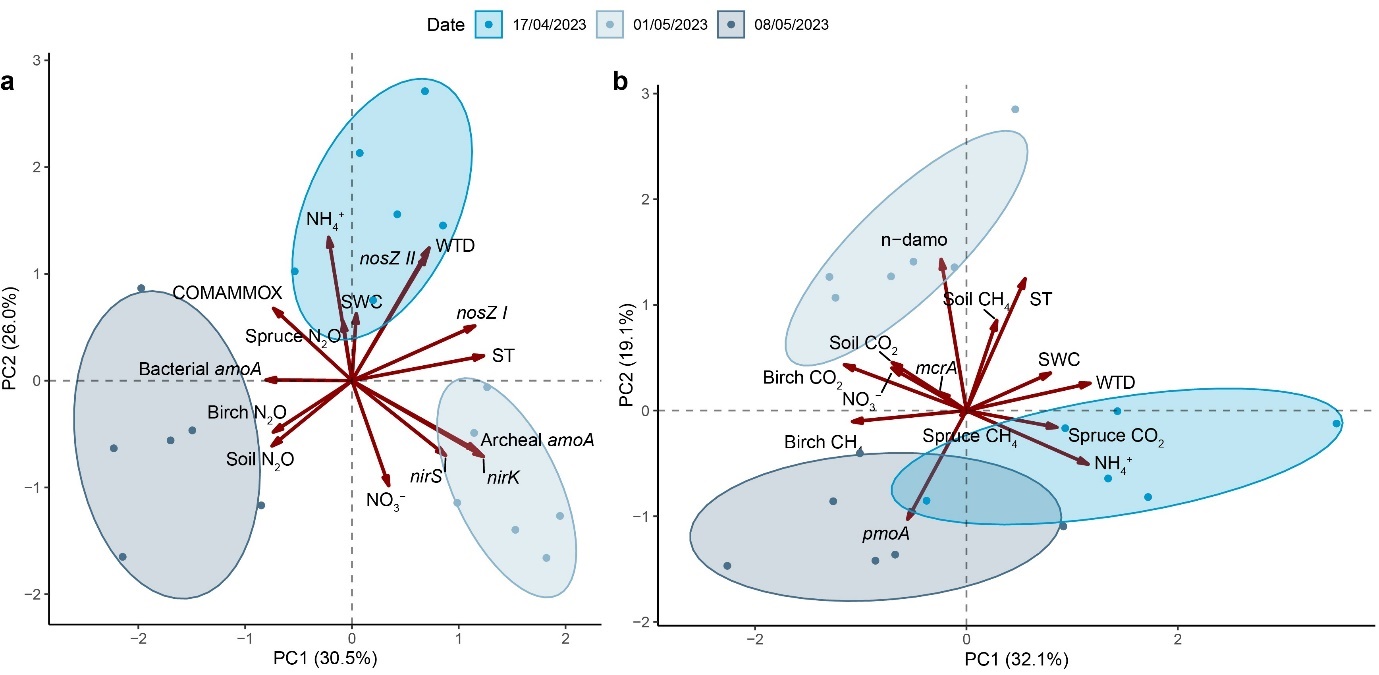
**

**Supplementary Fig. S8** Principal component analysis of the soil, birch and spruce GHG fluxes along with soil NO_3_ and NH_4_ content, soil water content (SWC), water table depth (WTD), soil temperature (ST), and functional genes’ abundance during the soil sampling days (17/04/2023, 01/05/2023, 08/05/2023). **(a)** PCA for the N cycle; **(b)** PCA for the CH_4_ cycle. Ellipses represent 95% confidence intervals for principal components on the different sampling days
